# The Impact of Dietary Supplementation of Whole Foods and Polyphenols on Atherosclerosis

**DOI:** 10.3390/nu12072069

**Published:** 2020-07-12

**Authors:** Abigail E. Cullen, Ann Marie Centner, Riley Deitado, Javier Fernandez, Gloria Salazar

**Affiliations:** 1 Department of Nutrition, Food and Exercise Sciences, Florida State University, Tallahassee, FL 32306, USA; ac12k@my.fsu.edu (A.E.C.); amc18ek@my.fsu.edu (A.M.C.); rd16k@my.fsu.edu (R.D.); jdf17c@my.fsu.edu (J.F.); 2 Center for Advancing Exercise and Nutrition Research on Aging (CAENRA), Florida State University, Tallahassee, FL 32306, USA

**Keywords:** polyphenols, atherosclerosis, whole food, fruits, vegetables

## Abstract

The purpose of this review is to highlight current research on the benefits of supplementation with foods with a diverse polyphenol composition, including fruits, vegetables, nuts, grains, oils, spices, and teas in blunting atherosclerosis. We searched PubMed for publications utilizing whole food or polyphenols prepared from whole foods in Apolipoprotein E (ApoE) or Low-Density Lipoprotein Receptor (LDLR) knockout mice, and identified 73 studies in which plaque was measured. The majority of the studies reported a reduction in plaque. Nine interventions showed no effect, while three using *Agaricus blazei* mushroom, HYJA-ri-4 rice variety, and safrole-2’, 3’-oxide (SFO) increased plaque. The mechanisms by which atherosclerosis was reduced include improved lipid profile, antioxidant status, and cholesterol clearance, and reduced inflammation. Importantly, not all dietary interventions that reduce plaque showed an improvement in lipid profile. Additionally, we found that, out of 73 studies, only 9 used female mice and only 6 compared both sexes. Only one study compared the two models (LDLR vs. ApoE), showing that the treatment worked in one but not the other. Not all supplementations work in both male and female animals, suggesting that increasing the variety of foods with different polyphenol compositions may be more effective in mitigating atherosclerosis.

## 1. Introduction

Cardiovascular Disease (CVD) is a collective term that applies to many different problems associated with the cardiovascular system (CVS), including atherosclerosis, coronary heart disease, arrhythmia, and hypertension. Atherosclerosis is a condition in which the arterial walls harden due to plaque accumulation in the neointima. The formation of the atherosclerotic plaque involves the migration of monocytes from circulation into the intima, where the inflammatory process allows their conversion into macrophages, which engulf oxidized low-density lipoprotein (oxLDL) particles, becoming foam cells. The recruitment of additional leukocytes further contributes to the accumulation of pro-inflammatory cells and plaque progression [1].

In just the United States (USA), the death rate attributed to CVD climbed significantly from the early 1900s into the 1980s; however, it has steadily decreased since the early 2000s [2]. This decline is likely caused by recent changes in lifestyle patterns, as well as the development of medications such as cholesterol-lowering statins [3]. As of 2017, risk factors that promote CVDs such as smoking, low physical activity, the consumption of high fatty food, high low-density lipoproteins (LDL) values, and hypertension have decreased [2]. Despite the reduction in known risk factors, CVD is still a leading cause of death in the US, and the American Heart Association (AHA) estimates that by 2035 nearly 50% of the adult US population will present some form of CVD [4]. Vaping has also emerged as a new risk factor for CVD and overall mortality, as new vaping devices, unregulated nicotine dosages, and diverse compositions of juices and flavors become increasingly popular [5]. The medical cost that will accrue with the increase in CVD prevalence is estimated to reach one trillion dollars, establishing the relevance of affordable nutritional interventions in reducing the health and monetary burden of CVDs.

As previously mentioned, one of the main risk factors associated with CVDs is the quality of food that people eat. The typical American adult eats what is characterized as a “Western Diet”—a collection of high fats, saturated fatty acids, and carbohydrates that promote a chronically inflamed metabolic state [6]. According to the 2015–2020 dietary guidelines recommended by the US Departments of Agriculture (USDA) and Health and Human Services (HHS), less than 20% of the population meet the dietary recommendations for vegetables, and less than 30% for fruits [7]. This is exacerbated by the fact that close to 90% of the population over-indulges in sodium, and 70% eat far above the recommended amount of saturated fats and added sugars.

The 2015–2020 guidelines recommend two cups of fruit per day for adults. While a reasonable recommendation, according to the CDC’s 2017 Morbidity and Mortality Weekly Report, only 1 in 10 Americans met this quota [8]. The USDA compared the average US consumption to the recommendations, and Americans fall short in their consumption of fruits, vegetables, and dairy, while eating more than the recommended amount of meat and grains [7]. Interestingly, fruit, one of the more palatable healthy options, falls 60% below the recommended daily intake. There are several reasons why Americans are not eating enough fruit. The prevalence of food allergies ranges from 2-10% in the US depending on the state [9]. Although less common, allergies to certain fruits such as strawberries [10] drive the population to avoid them. Many fruits, for example peaches, apples, and apricots, lead to irritation in the digestive tract due to the high fructose content, which is a concern for people suffering from irritable bowel syndrome (IBS) [11]. Additionally, with the increase in diets that restrict carbohydrates and sugars, such as the ketogenic diet, there may be reduced fruit consumption, as some, like bananas, are high in carbohydrates. Other considerations are price and shelf life, since fresh fruit is often more expensive than other options, such as frozen or canned fruit, and spoils more quickly. In fact, many Americans have been turning to fast food and frozen meals. The National Center for Health Statistics reported that between 2013 and 2016, 31% of low income families ate out on any given day; importantly, this percentage was the lowest between middle income (36.4%) and high income (42%) families [12]. One could argue that some families eating out may choose healthy options; however, Seguin et al. showed that consuming food away from the home lead to reduced fruit intake and increased body mass index (BMI) in males, but not in females [13].

This leads to an interesting problem, because fruits and vegetables have many benefits that should make them desirable. The USDA highlights the dietary fiber, low sodium, and high vitamin content in fruit and vegetables in their Dietary Guidelines for Americans [7], but many people do not know about the benefits of polyphenols, like anthocyanins or flavonoids, present in fruits and vegetables. Derived from phenylalanine, polyphenols consist of a variety of different structures, including aromatic rings of carbon, hydroxyl, and other compounds [14]. These compounds are important for scavenging reactive oxygen species (ROS) to reduce oxidative stress and promote anti-inflammatory signaling cascades. Thus, the dietary inclusion of these nutrients is critically important, especially in inflammatory diseases like atherosclerosis.

The poor dietary choices made by many individuals have profound pathological effects and thereon contribute to the development of diseases. This review will analyze current research on the effects of dietary supplementation with whole foods and extracts from various fruits, vegetables, grains, teas, oils, and spices on atherosclerosis in mouse models to highlight major gaps in the field. A major gap identified is the low inclusion of females in nutritional intervention studies. Only six studies use both males and females, and no study evaluated the effect of dietary interventions in estrogen levels, which protect females from CVD. Interestingly, not all dietary interventions that reduce plaque showed an improvement in lipid profile (reduced low-density lipoproteins (LDL), total cholesterol (TC), and triglycerides (TG) or increased high-density lipoprotein (HDL)), suggesting that the bioactive compounds in functional foods improve vascular health, preventing inflammatory molecules such as oxLDL from entering the arterial wall.

## 2. Polyphenols

### 2.1. Polyphenol Structure and Classification

Plants produce phytochemicals called polyphenols, which are formed by aromatic ring structures from the shikimic and acetate pathways. These pathways, which produce secondary metabolites, are important in plants because they respond to stress and confer protection from UV radiation and pathogens [15]. These pathways are not found in animals and humans; rather, in humans, polyphenols are considered xenobiotics or foreign substances [16]. Because polyphenols are xenobiotics, their bioavailability in humans is low. Polyphenols are found in fruits, vegetables, tea, red wine, coffee, cocoa, spices, and herbs. The specific composition of polyphenols gives these foods their characteristic color.

**Figure 1:**
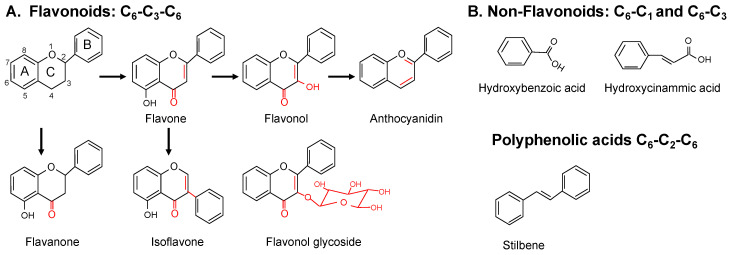
Structure of polyphenols. Polyphenols are divided into flavonoids (**A**) and non- flavonoids (**B**). Modifications to ring C and the addition of sugars to ring C (in red) allows for further sub-classifications.

Polyphenols can be divided into two major categories: flavonoids and non-flavonoids (Figure 1). There are approximately 8000 known polyphenols, and 4000 are flavonoids [14,17]. Flavonoids are the most extensively studied and can be broken down into additional groups, including anthocyanins, flavanols (flavo-3-ols), flavonols, isoflavones, flavones, and flavanonols. Flavonoids have a C6-C3-C6 phenyl-benzopyran backbone and a phenyl ring. Structural differences, such as a hydroxylation pattern and chromane ring (ring C) structure, allow for their sub-classification (Figure 1A). Flavonoids are found naturally as glycosides, meaning the organic compound portion (phenol) is bonded through its anomeric carbon or an oxygen molecule to a carbohydrate molecule through a glycosidic linkage (Figure 1A). The sugar portion is referred to as the glycone, and the non-sugar portion is the aglycone. Thus, the basic flavonoid structure is an aglycone or a phenol attached to a hydrogen.

An example of flavanols are catechins, which are found in berries, apples, tea, and cocoa [18,19]. While all catechins exert a strong antioxidant activity, the subtype epigallocatechin-3-gallate (EGCG) is characterized by having especially strong free-radical quenching properties [20].

Non-flavonoid categories include phenolic acids, polyphenolic amides, and other polyphenols [17] (Figure 1B). Phenolic acids include hydroxycinnamic acids and hydroxybenzoic acids. The base structure of the former is C6–C3, while the latter has a C6–C1 skeleton. They both contain one aromatic ring structure and side chains or groups. Caffeic acid (found in coffee, olive oil, and grains) and p-coumaric acid (found in peanuts, tomatoes, garlic, vinegar, and wine) are examples of hydroxycinnamic acids [21]. Examples of hydroxybenzoic acids include vanillic (in vanilla) [22] and gallic acid (a major metabolite of other polyphenols found in berries, walnuts, apples, and flax seeds) [18,23,24,25,26]. Polyphenolic amides include capsaicinoids (in chili peppers) [27] and avenanthramides (in oats) [28]. Other polyphenols include stilbenes, such as resveratrol (found in wine), curcumin (found in the spice turmeric), and ellagic acid (found in many fruits and nuts, including strawberries, raspberries, and walnuts).

Polyphenols commonly exert antioxidant and antimicrobial actions. For example, in food systems caffeic acid, p-coumaric acid, and rutin (a flavonoid found in apples and citrus fruits) were shown to confer antioxidant and antimicrobial activities, including heightened overall acceptance and antibacterial food preservation properties [21].

Just as the precursor to vitamin A, β-carotene gives carrots their bright orange hue and polyphenols often give color to the foods we consume. For example, anthocyanins give plants red, purple, and blue pigmentation, and are derived from flavonol [29]. Blueberries, blackberries, red raspberries, plums, and red cabbage are key foods rich in anthocyanins, which exist in a glycosylated form, cyanidin-3-glucoside being the main form in plants. The aglycone form is called anthocyanidin, and unlike anthocyanin does not contain a carbohydrate bonded at the 3 position. Furthermore, anthocyanins can be acetylated and anthocyanidin can be methylated, resulting in even more subcategories. The acidity of the environment influences the visible color of the anthocyanins. In an acidic environment, anthocyanins appear red and as the pH rises, they appear blue or purple. The nuances of anthocyanin and anthocyanidin pigmentation are extensively reviewed by Khoo et al. [30].

### 2.2. Polyphenol Metabolism

Phytochemicals such as polyphenols confer health benefits, such as protection against vascular inflammation, aging, and CVD [31,32]. Typically, the consumption of whole foods rich in polyphenols needed for a benefit range from 80 to 200 g, depending on the source [32]. Duynhoven et al. [33] and Cardona et al. [34] provide recent reviews depicting the role of polyphenols in human health, including thorough descriptions of their metabolism and mechanisms of action on the gut microbiota.

Polyphenols can take a number of routes through the human body. They can be absorbed by small intestine enterocytes and travel to the liver via portal circulation or be eliminated in the feces. From the liver, polyphenols can also travel back to the small intestine through bile before being excreted in the feces. Polyphenols can be also eliminated in urine [35].

Polyphenols consumed in their glycosylated form are poorly absorbed, and thus digestion is needed for proper absorption. Flavonoids undergo deglycosylation by β-glucosidases in the small intestine before they can be absorbed as aglycones [36]. Cardona et al. [34] reported that, on average, only 5–10% of dietary polyphenols are absorbed in the small intestine, while the remaining 90–95% are metabolized by bacteria in the large intestine. The absorption of polyphenols was evaluated in a review of 97 human polyphenol bioavailability studies by Manach et al. [37], while the importance of gut bacteria was observed using germ-free mice colonized with human flora [38].

After absorption, polyphenols undergo phase I and II biotransformations. Phase I reactions occur in enterocytes, while phase II reactions occur in enterocytes and hepatocytes. Phase I reactions include oxidative reactions such as decarboxylation, demethylation, dihydroxylation, while phase II reactions are conjugative, such as glucuronidation, acetylation, and sulfonation. Phase I and II reactions happen in the endoplasmic reticulum (ER) and require enzymes, such as cytochrome p450 enzymes (p450s), for phase I reactions, and UDP-glucuronosyltransferases (UGTs), sulfotransferases (SULTs), and glutathione S-transferases (GSTs) for phase II reactions [39,40]. UGTs facilitate the elimination of a number of xenobiotics such as drugs and phenolics—including natural polyphenols and pharmaceuticals [41]. The metabolites produced are water-soluble conjugates, derivatives of methyl, glucuronide, and sulfate, and can travel via systemic circulation to other organs and be excreted in urine. The large intestine plays a crucial role in the polyphenol metabolism, as it is responsible for the enzymatic breakdown of the large proportion of unabsorbed polyphenols. The colonic microbiota cleave glyosidic linkages and break down the heterocyclic polyphenol backbone. The importance of the gastrointestinal tract in polyphenol breakdown can be observed by tracing ellagitannins, which are hydrolysable tannins present in berries [42], pomegranates [43], walnuts [44], and oak-aged wines [45]. Ellagitannins undergo hydrolysis in the intestinal lumen, becoming free ellagic acid [46]. Ellagic acid travels to the colon and is metabolized to urolithins. These metabolites undergo one of two fates—absorption or excretion in the feces. If absorbed, they travel through the portal circulation to the liver and undergo phase II biotransformations before travelling via systemic circulation for tissue dissemination or urinary elimination.

Interestingly, the bioavailability of polyphenols can also differ dramatically. The extensive review by Manach et al. [37] concluded that gallic acid and isoflavones were the most well absorbed; catechins, flavanones, and quercetin glucosides were moderately absorbed; and proanthocyanins, galloylated tea catechins, and anthocyanins were the least well absorbed.

Gallic acid is an abundant phenolic compound in plant extracts in Mediterranean medicinal herbs, as reported by Mekinic et al. [47], and is also found in black tea [48] and berries (strawberries, blackberries, blueberries) [24]. Its metabolism is well described and its metabolites can be traced in the gut, blood, and urine [33]. The metabolism of gallic acid is described by Hodgson et al. [49].

### 2.3. Health Benefits of Polyphenols

Epidemiological studies suggest that polyphenol consumption mitigates several diseases, including CVDs [50] and cancer [51]. Polyphenols scavenge ROS, which contribute to a number of chronic-age related pathologies such as CVDs, cancers, Type 2 diabetes (T2D), and neurodegenerative disorders. Polyphenols can reduce inflammation, blunt hyperlipidemia and hyperglycemia, and improve metabolic syndrome.

Polyphenols increase the body’s endogenous antioxidant capacity by favorably influencing the rate-limiting enzyme that produces glutathione (i.e., gamma glutamylcysteine synthetase) [52]. The enhanced endogenous antioxidant capacity is through the nuclear factor erythroid 2-related factor/antioxidant response element (Nrf2/ARE) signaling pathway, leading to the upregulation of antioxidant enzymes, including superoxide dismutase (SOD), glutathione peroxidase (GPx), thioredoxin (Trx), reductase, and others, as reviewed by Ma [53].

In addition to improving chronic disease states, heightened endogenous antioxidants are beneficial for populations such as athletes, hastening muscle repair after exercise. Bowtell and Kelly published a recent review suggesting that >1000 mg of polyphenols from fruit each day for at least 3 days before and after exercise enhances muscle recovery through antioxidant and anti-inflammatory mechanisms [54]. Polyphenols also confer protection by enhancing vascular function through nitric oxide (NO)-mediated mechanisms.

Flavonoids reduce inflammation through blunted nuclear factor kappa B (NF-κB) intracellular signaling and nuclear translocation; the modulation of mitogen-activated protein kinase (MAPK) signaling [36]; and by the inhibition of ROS-producing enzymes, such as cyclooxygenases (COX), lipoxygenases, and inducible NO synthetase (iNOS) [55]. For example, blueberries rich in flavonoids and anthocyanins were shown to reduce COX2 in Ralph and William’s cell (RAW) 264.7 macrophages [56]. As a derivative of flavonol, it comes as no surprise that anthocyanins and their aglycone confer health benefits. They act as antioxidants and antimicrobials, scavenging free radicals produced by oxidative stress. Specifically, they can donate electrons to free radicals with unpaired electrons [30]. They can also scavenge free electrons through the attack of the hydroxyl group(s) of their B-ring and the attack of the oxonium ion on their C-ring [30].

DNA damage is associated with aging and chronic diseases, including degenerative diseases such as atherosclerosis and Parkinson’s. Grape seed extract (100 mg/kg), rich in flavonoids including gallic acid and catechins, has been shown to confer benefits by reducing age-associated oxidative damage in rats [57]. Compared to young rats, aged rats had reduced levels of endogenous antioxidants such as SOD, catalase, and GPx, as well as lower levels of vitamin C and E in the brain. The aged animals also had higher levels of lipid peroxidation due to free radical activity. Grape seed extract normalized antioxidants and lipid peroxidation in the brain of aged rats. In addition, green tea extracts (0.5 and 1 mg/kg) and a polyphenol found in green tea EGCG (2 and 10 mg/kg) increased the neuronal levels of the endogenous antioxidants SOD and catalase in a mouse model of Parkinson’s [58]. Furthermore, the same group observed that EGCG (0.01–5 μM) protected neuronal cells against oxidative stress by preventing stress-induced apoptosis through the restoration of PKC activity and the modulation of cell survival factors [59].

Impaired genomic stability after DNA damage can contribute to degenerative diseases [60]. Polyphenols can influence DNA stability in the presence of transition metals such as copper and iron. They do this through the Fenton Reaction by reducing transition metals to form hydroxyl radicals. This can be beneficial for tumor cells, as it results in apoptosis. However, it can also be mutagenic for normal cells. Polyphenols have been shown to be DNA damage-protective through other mechanisms. For example, they can prevent the formation of adducts, or segments of DNA bound to a cancerous chemical, a feature of carcinogenesis and early plaque formation [61]. In humans, the consumption of foods and beverages rich in polyphenols reduced oxidative damage to lymphocytic DNA. Examples include two separate studies which observed benefits with diets containing (1) onions (400 g) and tea (6 cups) for 2 weeks in diabetic patients [62], and (2) red wine (240 mL) for one month in combination with a high-fat diet [63]. The potential polyphenols with protective effects in these studies are flavonols—most likely quercetin, as it is found in onions and teas, and resveratrol, which is found in red wine. The former study also observed improved endothelial function.

Flavonoids can act as α-glucosidase inhibitors to favorably influence blood glucose and reduce CVD risk [64,65]. α-glucosidase is a carbohydrate hydrolase located at the brush border of the small intestine that cleaves α-1,4-glycosidic bonds to release α-glucose [66]. α-glucosidase inhibitors are suggested to blunt the progression of carotid intima-media thickness (IMT), a marker of atherosclerosis. IMT increases with age, a process that is accelerated in T2D, and α-glucosidase inhibitors confer protection by preventing carbohydrate digestion and postprandial hyperglycemia-induced hyperinsulinemia. Hyperglycemia contributes to atherosclerosis by the production of advanced glycation end-products (AGEs) and ROS and increased vascular inflammation through NF-κB activation. This topic has been reviewed extensively by others, including Mazzone et al. [67] and Brownlee [68].

Metabolic syndrome is a group of symptoms including hypertension, high cholesterol, hyperglycemia, and the accumulation of fat around the midsection [69]. Individuals suffering from metabolic syndrome are often overweight or obese and have a five-fold increased risk of T2D and two-fold increased risk of CVD [70,71]. The altered mechanisms leading to atherosclerosis and CVD in these individual include the increased levels of oxLDL, macrophage recruitment, and foam cell formation [72]. In addition, patients with metabolic syndrome have heightened plasma cytokines and adipokines, which contribute to inflammation. A study in rats suggests phenolic supplementation confers protection against metabolic syndrome. In this study, four groups received one of four phenolic acids (caffeic acid, gallic acid, ferulic acid, and protocatechuic acid) in 40 mg/kg body weight doses via gavage in combination with a high-fructose diet. All the treatments blunted the diet-induced metabolic syndrome. Specifically, polyphenols reversed insulin resistance, reduced blood sugar, improved altered lipids, and reduced inflammation and oxidative stress [73]. Furthermore, a synthetic flavonoid (S17834 derived from benzo(b)pyran-4-one) or resveratrol administration in a mouse model of metabolic syndrome prevented key heart disease pathologies, including left ventricle hypertrophy, interstitial fibrosis, and diastolic dysfunction [74]. S17834 exerted its effects through reducing oxidant-mediated protein modifications, ameliorating insulin resistance, and increasing the plasma concentrations of adiponectin. Adiponectin is an adipokine crucial to glucose homeostasis and fatty acid breakdown [75].

## 3. Atherosclerosis

### 3.1. Overview

Atherosclerosis is characterized by the accumulation of plaque within the walls of the arteries, a multilayered tissue comprised of the tunica adventitia, media, and intima. The outermost layer is the tunica adventitia, which is comprised of connective tissue and is crucial for arterial health. The dysfunction of this layer contributes to vascular disease, as was recently discussed by Tinajero et al. [76].

Inferior to the adventitia is the tunica media, consisting of numerous layers of vascular smooth muscle cells VSMCs that maintain vascular tone. Elastic lamina, composed of elastin and fibrillin-containing fibers [77], are barriers that surround the media and are arranged with VSMCs in a concentric fashion that provide the elasticity required by the aorta and other larger arteries [78]. Aging and chronic hypertension can damage the elastic fibers, causing arterial stiffness, which alters blood pressure dynamics [79]. When the elastic lamina wears out with age, stiffer collagen fibers take over, exacerbating arterial stiffness [79]. The elastic lamina contains fenestrations, which facilitate the transport of nutrients, connect cells within the interlamellar spaces [78], and mediate signaling between endothelial cells and VSMCs [80]. The remodeling of the lamina fenestrations plays a role in the progression of atherosclerosis. For example, in normal conditions the fenestrations allow small molecules to pass through the cell layers, but act as a barrier for high molecular particles such as LDL. If the size of the fenestrations were to increase, then LDL particles may enter the interlamellar space and initiate the accumulation of cholesterol, a cornerstone of atherosclerotic [81]. Alternatively, if the fenestrations were to decrease in size such as with chronic hypertension, the resulting lack of micronutrients such as calcium, which is crucial for contraction, could cause fibrosis or atheromatous medial degeneration [81].

The final innermost layer of the artery is the tunica intima or endothelium. This single layer of endothelial cells forms a semi-permeable membrane that regulates the trafficking of molecules, such as oxygen, water, and electrolytes. These cells are responsible for the production of NO, a potent arterial dilator [82]. Further promoting vasodilation, endothelial cells also secrete prostacyclin, a small lipid molecule that inhibits platelet aggregation [83]. The integrity of the intima is crucial for preventing the development of atherosclerosis, as endothelial cell damage decreases their selective permeability to water and electrolytes and allows larger particles such as LDL into the intima.

In atherosclerosis, cholesterol, lipids, calcium, and fibrin accumulate in the intima, forming a fatty streak, which expands over time to form a plaque. This results in arterial remodeling, the narrowing of the lumen, and a restricted blood flow, causing less oxygen and nutrients to be delivered to vital organs. The build-up of plaque can occur in arterial walls of the heart (coronary artery disease, CAD), the brain (cerebrovascular disease), the neck (carotid vascular disease), and the extremities (peripheral arterial disease, PAD) [84].

The migration of VSMCs contributes to plaque formation. During aging, states of inflammation, and with a high cholesterol diet, a new intimal layer emerges due to the migration of VSMCs from the media. This layer is called the neointima and contains not only VSMCs but foam cells, macrophages, cholesterol, and calcium crystals [85]. Together, these cells and molecules form a plaque, which may become fibrotic when extracellular matrix (ECM) components such as elastin and collagen are secreted by VSMCs. This matrix of ECM encapsulates a necrotic core formed of macrophages; LDL and oxLDL; foam cells; and synthetic, senescent, and apoptotic VSMCs. Within the core, proinflammatory M1 macrophages contribute to the secretion of interleukins (IL), chemokines, prostaglandins, and tumor necrosis factor-α (TNF-α), leading to increased inflammation [86]. In atherosclerosis, the secretion of ILs, such as IL-6, promote lesion development by turning on cell proliferation pathways regulated by Cyclin D1 [87,88]. With striking similarities to the building of bones, the calcification of the arteries occurs due to the secretion of inflammatory markers such as IL-1β, IL-6, and TNFα from macrophages [85]. Unsurprisingly, the more calcified the plaque is, the more stable it becomes. VSMCs also contribute to plaque stability by secreting collagen [89]. However, VSMCs can also become senescent and secrete inflammatory molecules (interleukins: IL-1α, IL-6, IL-8) and metalloproteases (MMP8, MMP9), driving plaque instability [90], which may lead to rupture and thrombus formation. Chemokines act as attraction signals for the immune system, promoting the migration of monocytes. Two important chemokines associated with atherosclerosis are stromal cell-derived factor 1 (SDF1, also known as CXC1L12) and macrophage migration inhibitory factor (MIF). The secretion of SDF1 leads to the oxidation of LDL, foam cell formation, and the interestingly increased stability of the fibrotic cap [91]. MIF is expressed highly by endocrine organs and other tissues, such as the lungs, gastrointestinal tract, and epidermis, and functions as an innate immune regulator by promoting the pro-inflammatory state of immune cells. MIF can potentiate signal transduction cascades by binding to CD74, an MHC class-II-associated invariant chain at the cell membrane. Specifically, MIF via CD74 activates extracellular regulated kinase 1 and 2 (ERK1/2) and prostaglandin E_2_ (PGE_2_) to induce VSMC proliferation and migration, respectively [92,93]. Prostaglandins further mediate inflammatory responses, primarily by stimulating rhodopsin-like 7-transmembrane-spanning G protein-coupled receptors (GPCRs), which are upstream of multiple pathways, influencing platelet aggregation, macrophage accumulation, and increased signal transduction by means of the second messengers cAMP, IP_3_, diacylglycerol (DAG), and intracellular Ca^2+^ [94].

Monocytes recruited to the endothelium express specific recruitment-facilitating receptors, including chemokine receptor 5 (CCR5), C-X3-C chemokine receptor 1 (CX3CR1), and C-C chemokine receptor 2 (CCR2) [95]. Monocytes contribute to plaque formation by the induction of mRNA and the protein expression of adhesion molecules such as vascular cell adhesion protein 1 (VCAM-1) and intracellular adhesion molecule 1 (ICAM-1) on the endothelium [96]. In the intima, monocytes differentiate into macrophages, as evidenced by their expression of cluster of differentiation 68 (CD68), a monocyte-lineage-specific molecule [97].

### 3.2. Polyphenols in the Cardiovascular System

Polyphenols play a role in reducing ROS, inflammatory processes such as monocyte adhesion and VSMC proliferation and migration, all of which are key events in atherosclerosis [98]. Specifically, polyphenols in animal models of atherosclerosis reduce oxLDL [99,100], a pathological instigator of endothelial dysfunction. In addition, S17834 reduced NADPH oxidase-dependent ROS production on endothelial cells and reduced atherosclerotic lesions in ApoE^−/−^ mice by 60% [101].

Anthocyanins have been reported to blunt smooth muscle cell proliferation through the perturbation of MAPK activity. For example, anthocyanin extracts (0.5–6 mg/mL) from the Hibiscus flower induced apoptosis in rat aortic smooth muscle cells (RASMs) through the activation of the p38MAPK and p53 pathways [102]. Apoptosis during atherogenesis blunts smooth muscle cell proliferation, migration, and subsequent lesion formation [102]. In addition, a major metabolite of anthocyanin, protocatechuic acid, has been shown to mitigate monocyte adhesion and blunt atherosclerosis in ApoE^−/−^ mice [103]. Bilberry extract, also rich in anthocyanins, has been also shown to favorably alter genes related to the development of atherosclerosis in ApoE^−/−^ mice [104].

Platelet-derived growth factor (PDGF) is a known inducer of VSMC migration [105], and red wine flavonoids (0.01–3% *v*/*v* wine) [106] have been shown to reduce VSMC proliferation and migration in RASMs. Specifically, red wine inhibits PDGFβ-associated signaling molecules such as Ras GTPase activating protein (Ras GAP), phosphoinositide 3-kinase (PI3K), the cytoplasmic SH2 domain containing protein tyrosine phosphatase (SHP-2), and phospholipid cleaving phospholipase (PLCγ). This prevents ERK1/2 phosphorylation, the induction of early gene transcription factors such as the proto-oncogene c-fos and early growth response 1 (Egr-1), which are associated with vascular lesion formation through the upregulation of VSMC proliferation and migration. In addition, the polyphenol pterostilbene, found in blueberries, has been shown to reduce VSMC migration by the attenuation of MMP2 [107]. MMP2 is a gelatinase that plays a role in the extracellular proteolysis of the basement membrane, allowing VSMCs to escape into the neointima during atherosclerosis progression [108]. Thus, polyphenols improve vascular function by reducing proliferation and migration and increasing the apoptosis of VSMCs.

Our group review the protective effects of polyphenols against vascular inflammation, aging, and CVD. Specifically, we described several mechanisms by which red grape juice, resveratrol, red wine extracts, and other polyphenols mitigate oxidative stress in the vasculature by inhibiting the expression and/or activity of NADPH oxidases, a major source of ROS [32].

## 4. Nutritional Interventions in Atherosclerosis in ApoE^−/−^ and LDLR^−/−^ Mice

The goal of this review is to highlight nutritional interventions using whole foods and whole food-derived extracts rich in polyphenols in reducing atherosclerosis. The search criteria for studies included ApoE^−/−^ or LDLR^−/−^, atherosclerosis, mice, polyphenols, and/or whole food supplementation from fruits, vegetables, grains, nuts, oils, teas, and spices. We focused on ApoE^−/−^ and LDLR^−/−^ because they are two of the most common models used to study atherosclerosis and show a consistent similarity with the results seen in human studies [109]. This analysis of current research has been broken down into four sections: fruits, vegetables, grains/nuts, and spices/teas/oils. Additionally, these sections are further broken down into subsections containing fruits with similar polyphenol profiles—for example, berries and grapes are rich in anthocyanins, while apples/litchi are rich in flavonols and prunes/plums are rich in cinnamic acids. Organizing the studies by conventional food groups and condiments/drinks facilitates the analysis of separate portions of the average diet.

**Figure 2:**
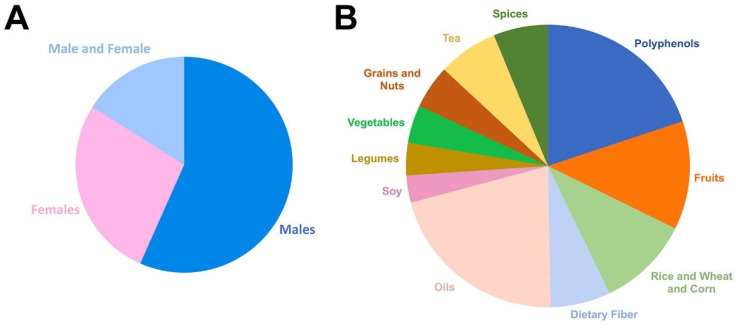
Sex and food categories of studies reducing plaque in mice. PubMed search for “mice/atherosclerosis/plaque”, including sex (**A**) and food categories (**B**).

A search in PubMED (8 June 2020) using the search criteria “mice/atherosclerosis” yielded 13,575 hits, excluding books, reviews, clinical trials, meta-analyses, and randomized controlled trials. Since many studies that have assessed the risk factors of atherosclerosis have not measured plaque, we refined our search to capture studies assessing plaque (mice/atherosclerosis/plaque), obtaining 3780 hits. From these studies, 2731 also included ApoE and 560 LDLR, while only 144 included both, suggesting that the majority of the studies have been performed in the ApoE^−/−^ model. We then assessed sex. Out of the 3780 studies, 1908 included male, 922 female, and 539 both male and female, indicating that more than 50% of studies only used male to assess atherosclerosis (Figure 2A).

Next, we focused on studies using nutritional interventions to reduce atherosclerosis. The refinement of the search “mice/atherosclerosis/plaque” including “food” or “polyphenols” yielded 313 and 32 hits, respectively. We then analyzed the different food categories represented in the “mice/atherosclerosis/plaque” search (Figure 2B) and found 32 for polyphenols, 20 for fruits, 17 for rice/wheat/corn, 11 for dietary fiber, 34 for oils, 5 for soy, 6 for legumes, 7 for vegetables, 8 for grains/nuts, 11 for teas, and 10 for spices/curcumin. From these studies, we collected 73 to analyze in detail. We focused on the mechanisms by which plaque was reduced, including the effect of the intervention in the lipid profile as well as the sex.

### 4.1. Fruits

#### 4.1.1. Berries

Berries are popular fruits consumed around the world fresh, baked into pies, and blended into smoothies. Berries are found in shades of red and purple because they are rich in anthocyanins [110]. One of the main deterrents of berry consumption is cost, as the affordability of berries is determined by the method of harvesting. In addition, berries tend to ripen more quickly than other fruits or grow mold, so they are often thrown out before they are able to be packaged and shipped. Buying frozen berries eliminates this waste; however, even those who eat frozen berries are still not reaching the recommended daily intake [111]. Frozen fruit consumption is on the rise, which is interesting because frozen fruits retain more vitamins than fresh fruits or vegetables due to flash freezing [111,112]. Our literature search yielded seven berry studies—including acai berry [113], blackberry [114], blueberry [115], lingonberry [116], hawthorn berry [117], bilberry [118], and black elderberry extract [119]—that matched our review criteria. Blackberries from the northern US, blueberry from the southern states such as Florida and California, and acai berry from South America are common in American cuisine, but the other berries are less known. Lingonberries can be found in northern parts of America, including the Pacific North West, however they are more common throughout northern Europe. The hawthorn berry originates from more temperate regions of the world, in particular, Turkey, which is one of the main producers of hawthorn berries [120]. Bilberries are also primarily found in northern Europe and have been rising in popularity because they contain one of the highest concentrations of anthocyanins [121]. These studies varied greatly in their experimental designs, which can be viewed in full in Supplementary Table S1, but one common method was adding the berry into the diet. Of the seven studies, only two (blueberry/acai berry) looked specifically at female mice and only one (blackberry) looked at both sexes; the remaining four studied males. All the supplements were administered to ApoE^−/−^ mice, so no genotype comparisons can be made. These studies were able to attenuate atherosclerosis to some degree, except for black elderberry, which promoted collagen deposition into the plaque, making it more stable and less likely to rupture which is beneficial [119]. Of note, our study on blackberry reduced plaque only in males, but not in females [114].

The effects on lipid profile markers, including TC, HDL, LDL, very-low density lipoprotein (VLDL), and TG, varied between treatments. Only the acai berry supplementation significantly improved HDL, but it had no effect on other lipid markers. Hawthorn berry and lingonberry reduced the TC and TG levels. Interestingly, both the blueberry and black elderberry treatments increased TC and LDL and showed no change in TG, however blueberry showed a trend to lower HDL levels. Black elderberry extract showed a trend to improve HDL. Blackberry and bilberry were the only treatments that showed no significant changes in any lipid profile marker.

One proposed mechanism by which these berries attenuated atherosclerosis is increased antioxidant capacity by the upregulation of glutathione reductase (GSR) and Trx 1 (blueberry) [115], SOD1 and SOD2 (blueberry and hawthorn berry) [115,117], and GPx1 and paraoxonase-1 (PON1) (acai berry) [113]. GSR and Trx 1 are part of a ROS-eliminating antioxidant mechanism through their activation of GPxs and peroxidredoxins, respectively [122]. GPx1 and PON1 reduce intracellular hydrogen peroxide and lipid peroxides [123], while SOD1 and SOD2 remove superoxide produced in the cytosol and mitochondria, respectively [124]. Additionally, the hawthorn berry reduced fatty acid synthesis and sterol regulatory element binding protein 1 (SREBP1), reducing circulating lipids, and increased GPx3; peroxisome proliferator-activated receptor *α* (PPARα), which regulates energy homeostasis by reducing the circulating TG levels [125]; and carnitine palmitoyltransferase 1 (CPT1), an enzyme essential for fatty acid β-oxidation in the mitochondria [126]. The mechanism by which bilberry reduced plaque was not elucidated, however it was not antioxidant in nature, and was most likely related to the anti-inflammatory properties of the anthocyanins found in the berry extract. Other proposed mechanisms include improved gut microbiota by lingonberry, which positively impacted cholesterol utilization by the liver, leading to reduced circulating lipids [116]. Blackberry, on the other hand, regulated oxidative stress by reducing the expression of the NADPH oxidase 1 (Nox1), an enzyme that produces superoxide and is upregulated during stress conditions, such as a high fat diet (HFD) [127]. Nox1 deficiency reduced atherosclerosis in ApoE^−/−^ mice [128]. Only males showed a reduced Nox1 expression and plaque in the aorta, an effect that was not observed in females.

In summary, the general trend for the attenuation of atherosclerosis by berries is due to reduced oxidative stress (improved antioxidant capacity and reduced Nox1). Not all the berries that reduced plaque improved the lipid profile, suggesting that reduced oxidative stress in the aortic wall is enough to improve cardiovascular health.

#### 4.1.2. Grapes and Pomegranate

Grapes and pomegranates have also been studied for their potential beneficial effects in mouse models of atherosclerosis and CVD. The most abundant polyphenols found in grapes are anthocyanins, flavanols, flavanals, and resveratrol [129]. Pomegranates are also rich in anthocyanins and flavanols, but additionally contain polyphenols such as punicalagin, which is unique to pomegranates, and ellagitannins, also commonly found in raspberries, walnuts, and teas [130,131,132]. We found a total of eight studies fitting the search criteria. They included two grape-based studies (Niagara grape extract with vitamin E and grape powder polyphenols) [133,134], one red wine grape pomace (the remnants from juicing grapes for wine) study [135], one dealcoholized-red and white wine study [136], a yellow rice wine study (the phenolic composition of yellow rice is comparable to red wine and is therefore included in this section) [137], and three pomegranate [138,139,140] studies fitting the search criteria. These eight studies implemented different methods of supplementation; further differences are presented in Supplementary Table S1. Grape studies utilizing the extract from grapes/seeds with vitamin E, the freeze dried grape polyphenol powder [134], the yellow rice wine extract [137], the pomegranate juice [138], and the byproduct (leftover fruit after juice is prepared) [139] were administered via drinking water. Red wine grape pomace and the dealcoholized red and white wine were added into the diet. Pomegranate polyphenols from the juice, byproduct powder, byproduct liquid, peels, arils (seeds), and flowers were given by oral gavage in gallic acid equivalents [140]. The pomegranate, dealcoholized-red and white wine, grape powder polyphenols, and grape extract with vitamin E were studied in ApoE^−/−^ mice. The yellow rice wine was studied in LDLR^−/−^ mice, and the red wine pomace was given to SR-B1 KO/ApoER61^h/h^ mice, which is a model of lethal ischemic heart disease [137]. Red wine grape pomace and Niagara grape extract with α-tocopherol (vitamin E) were looked at in males and females [133,135]. Two of the studies did not disclose the sex of the mice used [134,140]. The remaining four studies focused only on males, showing little representation of females in this branch of polyphenol research. Additional experimental details are provided in Supplementary Table S1.

The pomegranate byproduct (powder and liquid) [139,140], juice [138,140], and flowers [140] contributed to drastic changes in plaque after supplementation. Aviram et al. [140] reported a 44% reduction with juice, while Kaplan et al. reported a 17% reduction [138]. Aviram et al. additionally reported a 38% reduction with byproduct liquid, a 39% reduction with byproduct powder, and an astonishing 70% reduction with pomegranate flowers [140]. Rosenblat et al. determined that the pomegranate byproduct reduced lesion size by up to 57% [139]. Yellow rice wine reduced the lesion area by 40% in the 30 mg/kg per day group, which was the highest reduction even though this was the moderate dose-group, with 50 mg/kg being the high dose group [137]. Red wine grape pomace significantly reduced the lesion size; however an exact reduction in area was not given [135]. Grape powder polyphenols reduced the lesion size by 41%, and interestingly one of the mice in the study did not develop plaque in the aortic arch [134]. Of note, a concern was raised about the solubility of grape powder polyphenols in the drinking water used by Fuhrman et al., highlighting the importance of measuring the polyphenol content of the supplemented water the animals received [141]. Further, a reduction in plaque in the thoracic aorta was also observed with both red (62%) and white (30%) dealcoholized wine [136]. Additionally, the red wine reduced plaque by 16% in the aortic root. Interestingly two studies showed no change in plaque within the aorta, the pomegranate arils [140], and the grape extract with vitamin E [133]. The later however showed that the condition of the plaque (initial, intermediate, or advanced) improved with the treatment, with only 33.5% of the plaque being classified as advanced compared to 50% in the control group [133].

When assessing changes in lipid profiles, the effects of supplementation vary between studies. Only pomegranate flowers [137,140] and Niagara grape extract with and without vitamin E [133] were able to reduce TC and TG. The pomegranate flowers were additionally able to reduce the serum glucose levels. The pomegranate arils only reduced TG. Yellow rice wine significantly decreased TC and LDL; it slightly reduced TG, however it was not significant. The rest of the polyphenol groups, including the pomegranate byproduct power/liquid, and juice [138,140], grape powder polyphenols [134], red wine grape pomace [135], and dealcoholized wines [136], had no effect on any lipid profile marker. Unfortunately, lipid profile was not assessed in the pomegranate byproduct of the leftover fruit study [139], so it cannot be compared to the other studies. Importantly, while these studies show few effects on lipid profile, there were significant effects on the atherosclerosis lesion size.

The attributed cause of plaque reduction in size or severity was the reduction in macrophage oxidative stress, oxLDL uptake, and reduced lipid peroxide levels in many of these studies [134,138,139,140], which included all of the pomegranate and one of the grape studies. It was suggested that the peroxide levels are low due to the enhanced free radical scavenging ability of the pomegranate and grape polyphenols and that the polyphenols interfere with oxLDL binding to macrophage scavenger receptors, thus reducing cholesterol uptake [134,138,139,140]. Aviram et al. proposed that tannins provided protection against oxidative stress [140], while Kaplan et al. and Rosenblat et al. proposed that the PON and PON2 activities were increased, respectively [138,139]. Rosenblat et al. also found that the polyphenols in pomegranate significantly increased glutathione, which contributes to a reduced ability of cells to oxidize LDL cholesterol [139]. Peluzio et al. determined that Niagara grape extract with vitamin E increased the expression of LDL receptors in the liver, leading to increased hepatic cholesterol uptake, therefore reducing the circulating levels of cholesterol. They speculated that this reduction is one possible mechanism by which this extract reduced the progression toward more advanced plaque [133]. They also observed increased cholesterol and triacylglycerol excretion in the feces, furthering their position that improved cholesterol efflux was one of the main benefits of the treatment [133]. The dealcoholized wine [136] reduced plaque by reducing the adhesion molecules VCAM-1 and ICAM-1 and pathways including NF-κB, PI3K, mitogen-activated protein kinase kinase (MEK), interferon type 1 (IFN-1), and IL-1β. Specifically, by reducing IL-1β, the dealcoholized wines inhibited the MCP1 pathway, which mediates leucocyte adhesion to the endothelial wall, thereby reducing macrophage infiltration through the arterial wall and subsequent plaque formation.

Similar to berries, grapes and pomegranate show promising results attenuating atherosclerosis by reducing oxidative stress, as was shown through the reduction in macrophage oxLDL uptake and lipid peroxidation. Importantly, while they share similar polyphenol profiles, grapes were also shown to mitigate atherosclerosis through reducing inflammatory markers such as NF-κB and VCAM-1 where pomegranates did not change the inflammation status.

#### 4.1.3. Apples, Litchi, and Plums

Apples, litchi (also known as lychee), and plums are part of two fruit groups: pome and stone fruits, respectively. Apples are one of the most popular fruits on the market and are, according to the USDA, consumed ~50% of the time fresh or in juice. The primary polyphenol found in apples is quercetin, but it is also a source of vitamin C, flavonols, procyanidins, and phenolic acids [142]. Interestingly, the in vitro apple-derived procyanidin extracts inhibited lipoprotein secretion and the esterification of cholesterol in Caco-2/TC7 enterocytes [143]. Litchi are native to southern China but have spread throughout the tropical regions of Southeast Asia and even Australia [144]. They possess many polyphenols, including catechins, found in teas; anthocyanins; and procyanidins such as procyanidin B2, similar to apples [145]. Plums come in many varieties, originating from Eastern Europe, France, and China [146]. They have a slightly varied polyphenol profile compared to litchi and apples, including neochlorogenic and chlorogenic acid, which have been associated with improving antioxidant capacity and reducing LDL [147]. We found a total of five studies fitting the search criteria. There were three apple [148,149,150], one litchi [151], and one plum [152] studies. Two of the apple studies focused strictly on polyphenols from Fuji/Granny Smith apples and a mixture of apple polyphenols (not described) [149,150], and the other [148] compared polyphenols in the peel to the fiber from cider apples. The cider apple, Granny Smith apple, litchi, and dried plums studies incorporated the treatment into diets. Only the apple polyphenol study administered the treatment via gavage [150]. All of these studies utilized male ApoE^−/−^ mice, so no sex comparisons can be made. Additional experimental information can be found in Supplementary Table S1. Plaque was significantly reduced in all the five studies. It is important to note that the cider apple supplementation of both fiber and polyphenols and fiber alone produced the strongest reduction and were not different from each other (38.6% and 38.3%, respectively). Polyphenols on their own led to a 16.5% decrease, which leads to an important question: is fiber more important than the polyphenol content in fruits? This is something to consider for future research, as not many groups included the comparison of polyphenol to polyphenols with fiber in their experimental design. The content of polyphenols in the fiber extract was not analyzed; thus, the effect of fiber in atherosclerosis remains to be elucidated.

Only one of the studies had a significant beneficial effect on the lipid profile. Xu et al. reported that TC, TG, and LDL were reduced, while HDL was increased (~49%) by apple polyphenols [150]. In contrast, Gonzalez et al. and Auclair et al. observed that TC and TG were unchanged by their apple treatments [148,149]. Interestingly, unlike the other fruits, many possible mechanisms can explain the protective effects of apple, litchi, and plums in atherosclerosis. The apple studies attenuated atherosclerosis through a large variety of metabolic improvements, including increased PPARα, Nrf2, GPx, and SOD activity by apple polyphenols [150]; reduced plasma uric acid concentration (a byproduct of purine degradation that stimulates inflammation, leading to endothelial dysfunction [153]) by cider apple polyphenols [148]; and decreased circulating cholesterol in serum due to improved antioxidant capacity by Granny Smith apple peels [149]. Additionally, Xu et al. showed reduced VCAM-1 levels by apple polyphenols, similarly to dealcoholized wines [136]. The litchi study proposed that increased NO production attenuated atherosclerosis [151], while the plum study suggested that increased serum amyloid P-component (SAP) levels that reduce inflammation were the main cause for the reduced plaque accumulation [152].

Overall, apple, litchi, and plums are beneficial for atherosclerosis, showing again that plaque burden can be attenuated by lipid-independent mechanisms, as previously discussed in the blackberry, grape, and pomegranate studies. It is potentially through the improvements in oxidative stress by the reduction in adhesion molecules, inhibition of inflammatory pathways, and enhancement of antioxidant capacity that these fruits attenuate atherosclerosis.

### 4.2. Vegetables

#### 4.2.1. Roots and Gourds

Root vegetables are foods that are grown underground, acting as energy storage for the plant, meaning they are often high in carbohydrates/starches and fiber [154]. Common root vegetables include sweet potatoes, yams, turnips, parsnips, carrots, and onions. Gourd vegetables are formed above ground and are characterized by hard outer shells; their ranks include pumpkins, cucumber, squash, and melons such as watermelon and cantaloupe. The polyphenol composition varies from vegetable to vegetable, but some of the common polyphenols shared by root and gourd vegetables are carotenoids, which provide the yellow, orange, and red colors in some vegetables; anthocyanins; flavonoids; and ascorbic acid, which protect polyphenols from oxidation [155,156]. Few studies have been performed that analyze the effect of root or gourd vegetables on atherosclerosis. We found three papers which utilized very different protocols to assess anthocyanins from purple sweet potatoes [157]; an extract from Chinese yams [158]; and bitter melon [159], a member of the cucumber family popular in Eastern Asian cuisine. Anthocyanins from the purple sweet potato and bitter melon extracts were supplemented into the diet, while the Chinese yam extracts were administered via gavage. All three studies were conducted only in male ApoE^−/−^ mice, so again no sex comparisons can be made, and all mitigated atherosclerosis. Supplementary Table S2 provides further detail on the experimental design.

In terms of lipid profile changes, only the Chinese yam study showed significant changes, with an increase in HDL and reductions in TC, oxLDL, and C-reactive Protein (CRP) in plasma. Purple sweet potato and bitter melon had lesser effects on lipid profiles, with only bitter melon conferring a significant reduction in TG levels. These observations are in agreement with data from other foods such as blackberry, bilberry, apple, pomegranate, grapes, and wine polyphenols, in which reduced atherosclerotic plaque was mediated by lipid profile-independent mechanisms.

It is hypothesized that, in studies showing no changes in lipid profile [157,159], protection against plaque accumulation was mediated by reduced VCAM-1. Additionally, purple sweet potato anthocyanins lowered thiobarbituric acid in the liver, an oxidative stress marker associated with lipid peroxidation. Both bitter melon and Chinese yam treatment measured macrophage content within aortic plaque. While the bitter melon study shows reduced macrophage content, the Chinese yam study showed a marked increase in macrophages. Although surprising, these macrophages showed a reduction in IL-6, suggestive of macrophage activity inhibition within the plaque [158].

Root and gourd vegetables have not been studied enough to show a full picture of how they alter atherosclerosis, however the research that has been carried out shows that they are promising sources of future research. Their protection against atherosclerosis is likely through unknown lipid-independent mechanisms acting on chronic inflammation pathways stimulated in atherosclerosis.

#### 4.2.2. Cruciferous and Salad Vegetables

Cruciferous vegetables (broccoli, cauliflower, cabbages) and salad vegetables (leafy greens such as chicory and spinach) will be compared together due to nutritional similarities (iron, vitamin C, vitamin A) [160,161]. There are, of course, a myriad of polyphenols found in these vegetables, but some of the most abundant are quercetin, kaempferol, isorhamnetin, and a variety of caffeic acids [162]. The search yielded four papers that assessed plaque in ApoE^−/−^ mice. One studied chicory [163], a vegetable commonly found in the Mediterranean diet; one studied anthocyanins from red Chinese cabbage [164]; and two studied quercetin [165,166]. All four of the papers only studied the effects of treatment in male ApoE^−/−^ mice, so no comparisons can be made between sexes. Further details are provided in Supplementary Table S2. All four studies were reported to reduce plaque.

A lipid profile analysis showed that red Chinese cabbage (based on whole food/extracts) had beneficial effects, specifically by reducing the TC and LDL/VLDL levels [164], while the chicory study showed reduced cholesterol in the aorta [163]. This study did not measure lipids in circulation. Interestingly, the effects of quercetin reported by Loke et al. [165] did not alter the lipid profile even after 20 weeks of treatment. The other quercetin study by Shen et al. [166] showed that, after 14 weeks, treatment with 1.5 mg/d (as opposed to 1.3 mg/d by Loke et al.) significantly reduced TC and TG.

Chicory was reported to reduce plaque by lowering TC and activating ATP-binding cassette transporter 1 (ABCA-1) and ATP-binding cassette sub-family G member 1, which are pivotal players in HDL-dependent cholesterol efflux, to improve cholesterol efflux [163,167]. Anthocyanins from red Chinese cabbage reduced VCAM-1 and improved antioxidant capacity and lipid metabolism [164]. The atheroprotective mechanism of quercetin hypothesized by Loke et al. [165] was attributed to a reduction in leukotriene B4, LDL oxidation, and improvements in NO availability by the increased eNOS activity and heme-oxygenase-1 (HO-1) [165]. Leukotriene B4 is an agonist of inflammatory responses involving TNF-α and interleukins and the recruitment of monocytes and neutrophils [168]. Shen et al. also reported an increase in eNOS activity and HO-1 as the primary mechanism by which quercetin reduced atherosclerosis [166]. HO-1 mediates the rate-limiting step of heme degradation and prevents atherosclerosis by its effect on bilirubin, which reduces lipid peroxidation, but HO-1 also inhibits lesion development in LDLR^−/−^ mice in a lipid-independent manner [169].

Like the root and gourd vegetables, not many studies have investigated the benefits of cruciferous vegetables on atherosclerosis. The limited research has shown, though, that the whole food and its polyphenols are atheroprotective. Importantly, it seems that part of the benefits of these foods are mediated by quercetin, one of the primary polyphenols in cruciferous vegetables, which promoted eNOS activity and HO-1 expression to reduce oxidative stress and plaque accumulation.

#### 4.2.3. Soybeans

Soybeans are from the legume family and have risen in popularity over the last several years as they provide plenty of protein, carbs, and fats to vegan and vegetarian diets. They are set apart from other plant proteins, as they are considered a complete protein, containing all nine essential amino acids. After being cultivated in Southeast Asia, soybeans were brought to Eastern Europe and America, where they have been cultivated so much that there are now two types of soy beans—domesticated and undomesticated, which is found in the wild [170]. Soy is rich in polyphenols, including anthocyanins, gallic acid (a metabolite of polyphenols), and isoflavones [171]. Little has been studied on the direct effects of soybeans and their derivatives on atherosclerosis, however our literature search found three studies centering around soy. The papers included phytochemicals from soybeans (soyasaponin A1 and A2) [172], soy germ vs. tomato powder [173], and soymilk [174]. The soyasaponin A1/A2 and soy germ and tomato powder were given to ApoE^−/−^ mice, while the soymilk was given to LDLR^−/−^ mice. All three studies utilized male mice, so no sex comparisons can be made. The soyasaponins and soy germ were supplemented in the diet, and the soymilk was administered via gavage. Extended experimental details can be found in Supplementary Table S2. The soyasaponins and soymilk studies significantly reduced plaque size in the aorta, however neither soy germ nor tomato powder had an effect. Additionally, soy germ and tomato powder were unable to alter the lipid profile [173]. Soyasaponin A1 proved extremely beneficial by reducing TG, TC, and LDL and increasing the HDL levels [172]. Soyasaponin A2 had similar effects on the lipid profile, however it did not alter the HDL status. Soymilk shared similar results, with reductions in TC, TG, LDL, and VLDL and increased HDL. Importantly, the TG and VLDL in soy milk-treated animals were returned to the control basal levels [174].

Plaque reduction induced by soyasaponin and soymilk treatments was attributed to the concurrent decrease in hypercholesterolemia and inflammation. Soymilk reduced CRP and CD40L, which enhance phagocytosis and initiate the immune response, and prime T and B cells to activate the immune response, respectively [175,176]. The soyasaponins reduced TNF-α and MCP-1 and increased cholesterol efflux, however the precise mechanism was not elucidated.

Soy and its polyphenols show promising future research in attenuating atherosclerosis. This is particularly important to follow, as soy has been increasing in popularity over the last several years.

#### 4.2.4. Mushrooms

Mushrooms are the fruits of different fungi that grow in dark and humid places. These fungi have been used for thousands of years as a source of food and for their medicinal properties [177]. They have been shown to be strong potentials for prebiotics due to their high content of polysaccharides such as chitin and glucan, making mushrooms popular for treating gastrointestinal ailments [178]. There are many edible mushrooms; some of the most popular are portobello, button, oyster, cremini, and shiitake. Specific polyphenol compounds vary from mushroom to mushroom, but the total polyphenol content and antioxidant capacity are consistently high [179]. One of the most impactful components is a naturally occurring amino acid, ergothioneine, which, when taken up by endothelial cells, can reduce endothelial dysfunction and oxidative stress [180]. Our literature search returned four studies investigating the effects of edible mushrooms on atherosclerosis. The mushroom studies included *Grifola gargal* Singer (gargal) [181]; portobello and shiitake [182]; erinigi, maitake, and bunashimeji (EMB) [183]; and *Agaricus blazei* (blazei) [184]. The gargal, EMB, and blazei mushrooms were given to ApoE^−/−^ mice, and the portobello and shiitake were given to LDLR^−/−^ mice. All the studies used male mice except blazei, which did not specify sex. The delivery was different between the studies. The gargal extract was administered by IP, while the EMB, blazei, portobello, and shiitake were mixed directly into the diet. Interestingly, two studies, gargal and EMB, gave animals a normal chow diet. The gargal-treated animals were infused with Ang II via osmotic minipump to induce atherosclerosis. Plaque was significantly reduced by the gargal, portobello, shiitake, and EBM treatments. Interestingly, the blazei mushrooms increased plaque, which was thought to be in response to enhanced leukocyte migration and inflammation. Full experimental details can be found in Supplementary Table S2.

The only changes to lipid profile were seen with the portobello, shiitake, and bunashimeji mushrooms. Both portobello and shiitake reduced TC, TG, and LDL, but they differed in their effects on HDL; portobello caused no change and shiitake decreased HDL. Bunashimeji only reduced TC, while erinigi and maitake only reduced TC during the 6th week of the trial (out of 10). There was no effect on lipid profile with the blazei mushroom, and the gargal study did not assess the lipid profile.

There was no consensus of the specific mechanism that promoted reduction in atherosclerosis with mushroom supplementation. The gargal improved inflammation by lowering the circulating granulocytes, important for inflammation responses, and normalizing regulatory T cells, which prevent the excessive response of other T cell subtypes, specifically Th1 and Th17. The shiitake and portobello reduced TNF-α, circulating lipids, and VCAM-1 expression. Of the EMB mushrooms, bunashimeji reduced plaque to the greatest extent. Mori et al. hypothesized that bunashimeji, which enhanced the fecal excretion of cholesterol, was the cause of the lipid profile improvement.

Overall, mushrooms are promising prospects for further atherosclerosis research. They could become a popular option for treating atherosclerosis, as they are free of cholesterol, low calorie, and have a large variety of types [185]. There are mushroom powders on the market which could be an alternative consumption method, allowing people to get the nutritional benefits of the mushrooms without having to eat them whole.

### 4.3. Grains and Nuts

#### 4.3.1. Corn, Rice, and Wheat

Corn, rice, and wheat are common carbohydrate staples typically cut out in trending diets, such as the paleolithic (paleo), whole30, and ketogenic diets [186,187]. Whole30 is a very restrictive diet that cuts out grains, legumes, sugar, alcohol, dairy, and soy. Grains are avoided on Whole30 as a way to reduce inflammation caused by proteins, such as gluten, while the ketogenic diet prohibits grains because their consumption will reintroduce carbohydrates to the diet, altering the metabolic ketosis state which is desired by those on the diet. The paleo diet is focused on whole foods, instead of processed foods. Only the paleo diet has been studied rigorously, and was shown to cause on average a 9% loss in weight; the Whole30 and ketogenic diets have not been studied in detail [186]. Anton et al. show that, when evaluated for long-term vs. short-term weight loss, these diets only promote larger weight losses when utilized in the short term [186]. Unfortunately, this effect encourages the cycling of diets in many who are trying to lose weight. The constant flux of dietary restrictions, or “yo-yo” dieting, is not beneficial for the metabolic state of the body [188]. Eating patterns that constantly change show that, in parallel with issues in self-image and peer pressure from media, this weight cycling promotes cardiometabolic disease, which can lead to obesity, diabetes, and even cancer [188,189]. Since obesity and diabetes are diseases that drive inflammation, they increase the risk of atherosclerosis. It is therefore important to educate the public that, while reducing the intake of carbohydrates by cutting out rice, wheat, and corn may lead to immediate (but short lived) weight loss, the yo-yoing effect on the metabolic system is deleterious. What is less known about rice, wheat, and corn is that these foods are rich in phenolic acids and flavonoids and, when in combination with legumes, for example white rice and black beans, a complete protein meal can be achieved [190,191]. In our search, we found a total of ten papers fitting this review’s criteria, including dasca-flint corn fractions (aleurone, germ, and endosperm) [192]; anthocyanins from black rice [193]; red yeast rice [194]; rice bran extract [195]; rice protein isolate [196]; wild rice [197]; the HYJA-Ri non-glutinous rice variety [198]; cereal fibers, including oat and wheat bran [199]; yellow dent corn and hard red spring wheat bran [200]; and major safflower, a crop popular for its seed oil grown in the Great Plains [201].

Of these ten papers, seven studied ApoE^−/−^ [193,194,195,196,199,200,201] and two, studying wild rice [197] and dasca-flint corn fractions [192], looked at LDLR^−/−^ mice. HYJA-Ri non-glutinous rice was the one study to utilize a double knockout of both ApoE and LDLR [198]. The only study that investigated female animals was the rice protein isolate [196]; the remaining nine studies utilized male mice. All the studies supplemented the treatments into the diets and all reported reductions in atherosclerotic plaque, except the yellow dent corn/hard red spring wheat bran and the HYJA-Ri rice variety, which increased plaque. Further detail of experimental design is included in Supplementary Table S3.

Black rice anthocyanins, rice bran, major safflower, flaxseed, oat fiber, and the red yeast rice reduced TC. The black rice anthocyanins, rice bran, and red yeast rice also reduced LDL/VLDL cholesterol. Only black rice anthocyanins and rice bran were able to additionally reduce TG and improve HDL cholesterol. Rice protein isolate had no effect on TC or HDL, but oxLDL was reduced [196]. The corn fraction of aleurone and germ reduced TC but had no effects on HDL, LDL, or VLDL. The endosperm had no impact on any lipid profile, and none of the three fractions impacted TG. The wild rice study only measured lipids in fecal samples, which were significantly increased by wild rice and phytosterols, which leads to the assumption that circulating lipids were decreased, but this cannot be confirmed without further testing [197]. Yellow dent corn and hard red spring wheat bran had no effect on lipid profile markers [200]. The lipid profile was not assessed in the HYJA-Ri rice study [198].

In terms of mechanisms, there was no consensus between the studies. However, several including flaxseed, major safflower, corn fractions, and red yeast rice attribute their supplementation’s benefits to reductions in IL-6 and VCAM-1, reductions in lipid peroxides and TC, improved gut microbiota, and improved lipid metabolism, respectively. Black rice improved plaque stability by inhibiting MMP1 activity and tissue factor, known for its role in blood coagulation and iNOS activity [193]. Rice bran increased the apoptosis of mononuclear cells through the upregulation of p53; p16; and the mitochondrial apoptosis regulator, bax [195]. Moghadasian et al. did not propose a mechanism by which wild rice reduced atherosclerosis, however they proposed that increased lipid excretion was involved [197]. Oat fiber and wheat bran were suspected to reduce plaque by dampening the inflammatory response initiated by the NOD-, LRR-, and pyrin domain-containing protein 3 (NLRP3) inflammasome, which is activated by NF-κB, toll like receptor 4 (TLR4), and myeloid differentiation primary response 88 (MyD88) signaling [202].

While there was no consensus on the specific mechanisms by which these foods attenuated plaque, it seems to be through a reduction in inflammation, associated with reduced levels of interleukins and inflammasome activity.

#### 4.3.2. Nuts and Seeds

Nuts are fruits of plants surrounded by a large, hard, inedible shell, and seeds are reproductive parts of plants that can, if planted, turn into another plant. Most nuts are the seeds of many plants, such as walnuts, cashews, and pistachios. Whether or not the botanical classification is a nut or a seed, many people consume these foods at similar rates or in similar ways. The CDC reported that between 2009 and 2010, 38.2% of adults given the National Health and Nutrition Examination Survey (NHANES) ate some form of nut or seed on average day [203]. Many people consume nuts and seeds roasted or in the form of a nut/seed butter. Nuts have many health benefits, such as improving the lipid profile and reducing endothelial oxidative stress and inflammation due to their polyphenol content [204]. Nuts and seeds are also incredible sources of folate, tocopherols, polyphenols, calcium, and other nutrients [205]. We found four studies using walnuts [206], tree nuts (macadamia and pecan) [207], a nut mix (50% walnut, 25% almond, and 25% hazelnut) [208], and flaxseed [209]. The tree nuts, nut mix, and walnut studies were conducted in ApoE^−/−^ mice, and the flaxseed study used LDLR^−/−^ mice. The tree nut and walnut studies were performed in males. The flaxseed one utilized females, while the nut mix study included both males and females. Experimental details can be found in Supplementary Table S3. All the studies reduced plaque, except for the nut mix, in which a significant impact on plaque was observed only in females. Male mice in this study showed either significant reductions in plaque or significant increases in plaque, leading to inconclusive results on the actual effect on plaque [208]. The tree nuts reduced plaque in the brachiocephalic artery, but there was no effect on plaque accumulation in the aortic arch [207]. Additionally, it is important to note that in the walnut study [206], whole walnuts but not walnut oil reduced plaque, providing evidence of the benefits of whole foods.

While the tree nut study did not assess lipid profiles, the walnut, flaxseed, and nut mix studies reported interesting finds. The walnut study reported that only whole walnut but not walnut oil was capable of reducing circulating TC and TG and liver TG [206]. Flaxseed supplementation only reduced TC and plasma saturated fatty acids, and no other lipid markers were tested [209]. In the nut mix study, treatment reduced LDL/VLDL in both males and females, TC was only reduced in males, and no change in HDL was observed in either sex [208].

There were varying proposed mechanisms for the cause of atherosclerosis attenuation. While flaxseed was able to reduce TC, the proposed mechanism was thought to be lipid-independent and caused by a reduction in IL-6, VCAM-1, macrophage marker M3/84 (mac-3), and proliferating cell nuclear antigen (PCNA). PCNA binds to DNA and promotes the activity of DNA polymerase, a benefit to cells, such as synthetic VSMCs, which proliferate rapidly within atherosclerotic plaques [210]. With the complex composition of polyphenols found in the macadamia and pecans, it could be due to an increase in antioxidant capacity or lipid profile normalization. Further testing would need to be performed to fully understand the mechanism by which these nuts reduce plaque. The walnut paper proposed that the benefits could be mediated by the reduction in CD36-presenting macrophages. CD36 is a receptor for thrombospondin-1 that is involved in immunity and fatty acid signaling and was reported to be localized in the atherosclerotic plaque [207,211]. The nut mix attenuated atherosclerosis by reducing LDL and increasing PON2 [208], as seen in the pomegranate byproduct study [139].

Nuts and seeds have not been studied extensively; however, they are a good candidate for future research. They are affordable, bioavailable, and appetizing snacks filled with polyphenols. The only limitation to this potential treatment is food allergies, which are becoming more common.

### 4.4. Oils, Spices, and Teas

#### 4.4.1. Oils

It is commonly thought there are healthier oils, such as olive oil, which is high in monounsaturated fatty acids [212], and unhealthy oils, which are high in saturated fatty acids, such as tropical oils including palm and coconut oil [213]. While they are not all created equally, oils do provide a variety of polyphenols that could beneficially impact the CVS. In our literature search, we found five studies that use extra virgin olive oil (EVOO) with and without green tea polyphenols [214], seal oil [215], pequi oil [216], or perilla oil [217], and one that compares the effects of palm, echium, and fish oil [218]. The EVOO with green tea polyphenols and perilla studied only male ApoE^−/−^, and EVOO with seal oil was supplemented in male and female ApoE^−/−^ mice. Pequi oil was studied in female LDLR^−/−^ mice. Importantly, the palm/echium/fish oil study utilized both ApoE^−/−^ and LDLR^−/−^ females. Further details on experimental design can be found in Supplementary Table S4.

The effects on plaque accumulation were not uniform in these studies. EVOO with seal oil reduced plaque within the aortic arch and descending aorta in female mice, however it only reduced plaque in the descending aorta in male mice [215]. EVOO with green tea polyphenols had significant effects on plaque reduction throughout the entire aorta of about 20%, compared to EVOO alone (11%) [214]. Perilla oil significantly reduced fatty streak lesion formation in the aortic sinus [217], but not in the aortic root, and even promoted more advanced lesions, consistent with the increase in TC, LDL/VLDL, and TG by this oil. The plaque in animals treated with pequi oil seemed to be more stable due to higher levels of collagen and a thick fibrotic cap. Pequi oil reduced the plaque area in the descending aorta. Interestingly, in the palm, echium, and fish oil study, the treatments significantly reduced plaque accumulation within the aortic root in LDLR^−/−^ mice but had no effect in ApoE^−/−^ mice.

Regarding lipid profile, EVOO and seal oil reduced TC in both male and female mice [215], and with the perilla, echium, and fish oil treatment, LDL/VLDL were reduced and HDL was improved in LDLR^−/−^ mice [218]. Interestingly, these beneficial effects were not seen in ApoE^−/−^ given the same treatment. In fact, fish oil increased their plasma cholesterol. These observations highlight the need to understand the differences between the murine models used in CVDs so that a more comprehensive mechanistic explanation can be found. Major differences between the ApoE^−/−^ and LDLR^−/−^ models can be found in a review by Getz et al. [219].

Similarly to the fish oil treatment in ApoE^−/−^ mice, pequi oil increased TC, LDL/VLDL, and TG and had no effect on HDL [216]. EVOO with and without green tea polyphenols reduced oxLDL and lipid peroxidation; however, this study did not measure TC or any other lipid profile marker [214].

The majority of these papers [214,216,218] state that the protective effects in atherosclerosis are mediated by antioxidant mechanisms of the omega-3 fatty acids found in the oils. In the palm, echium, and fish oil study, the authors further speculated that the omega-3 fatty acids contributed to the reduced circulating Ly6C^hi^ monocytes, which are known to be found in atherosclerotic plaques in the LDLR^−/−^ murine model [218]. EVOO with green tea polyphenols altered the macrophage cholesterol metabolism, which yielded a reduced uptake and increased HDL-mediated efflux likely through the control of ABCA-1, as cited by the chicory study [163]. The seal oil and EVOO study discussed different mechanisms for plaque reduction, primarily through reduced lipid peroxidation [215]. Importantly, the pequi oil is significantly higher in saturated fatty acids, which is the predicted cause of the enhanced lesion status in the aortic root. However, the reduction throughout the rest of the aorta was predicted to be due in part to reduced oxidative stress and oxLDL by an antioxidant mechanism [216].

Overall, supplementing oils into the diet, especially olive oil, reduces atherosclerotic plaque; the primary mechanism of interest for this result is the improvement of antioxidant capacity.

#### 4.4.2. Spices and Herbs

Several other reviews [220,221,222] have highlighted the importance of spices and their roles in attenuating CVDs. A variety of polyphenols can be found in spices; the most commonly found are phenolic acids and flavonoids [223]. For example, turmeric is a spice commonly used in Indian cuisine that is packed with curcuminoids, which provides the intense yellow pigment to the spice [223], and chocolate is rich in catechins and procyanidins [224,225]. Our review search yielded eleven studies with polyphenols from spices and herbs that met our criteria. The results include two papers testing curcumin (a polyphenol found in turmeric) [226,227]; one paper testing cacao polyphenols (specifically procyanidin B2, catechin, epicatechin, procyanidin B5, procyanidin C1, and cinnamtannin A2) [228]; a herbal mix including *Artemisia iwayomogi* Kitamura and *Curcuma longa* Linne (artemisia and turmeric; AT) [229]; and bee pollen, which primarily contains phenolic acids and flavonoids, but the composition changes depending on which flowering plants bees visit [230]. This specific pollen was collected from dandelion, European raspberry, rapeseed, buckwheat, linden, and clover plants [230]. Another study used star anise [231], a plant similar to a licorice root, which contains polyphenols such as trans-anethole and caryophyllene (a terpenoid derivative) [232]. The search additionally found studies utilizing safrole-2’,3’-oxide (SFO) [233] and caffeic acid phenethyl ester (CAPE) [234], which are specific polyphenols found in various spices and herbs, including thyme, oregano, basil, sage, and turmeric. CAPE is a relative of 3,4-dihydroxycinnamic acid and can also be found in honey [234]. SFO is mainly found in sassafras oil as well as black pepper, star anise, and nutmeg [233]. Additionally, there were studies using polyphenols from herbs used in popular Chinese medicine, including β-elemene [235], found in curcuma species; Ginsenoside Rb1 (Rb1) [236], found in ginseng; and geniposide [237], found in the fruits of gardenia trees. Eight of these studies [226,227,229,231,233,234,235,236] looked at male mice and only one, bee pollen [230], studied females. Two studies that focused on cacao polyphenols and geniposide from gardenia fruit [228,237] did not disclose the sex of the mice used. ApoE^−/−^ mice were used for all the studies, so no genotype comparisons can be made. Several delivery methods were used in these studies. The curcumin (Zhou et al. [227]), cacao polyphenols, bee pollen, CAPE, and AT were supplemented into the diet. The star anise, curcumin (Zhao et al. [226]), and geniposide were administered by gavage, and it is likely that β-elemene was administered this way, but it was not explicit. SFO and Rb1 were injected via IP. Additional experimental information can be found in Supplementary Table S4. A plaque analysis indicated that all of the treatments reduced plaque, except for the SFO supplementation [233].

The majority of the treatments attenuated circulating TC [226,227,229,230,231,236,237], with one curcumin study [227] also indicating that the cholesterol in the aorta was significantly reduced. There was a similar trend in studies also showing that LDL/VLDL cholesterol was reduced [226,227,229,231,236,237]. The bee pollen study however did not measure these lipids, instead indicating that oxLDL was reduced by 59% [230]. TG was not measured in all of the studies, but curcumin via gavage [226], AT [229], and Rb1 [236] showed reductions. The curcumin in the diet study along with geniposide showed no reductions in TG [227,237]. Curiously, the same three studies that reduced TG (curcumin via gavage [226], AT [229], and Rb1 [236]) also increased HDL. The β-elemene and cacao polyphenols were unable to change any lipid profile marker [228,235]. SFO was the only treatment that showed negative effects on the lipid profile, yielding increases in TC, LDL, and TG [233], which is consistent with the lack of effect on atherosclerosis. As before, some studies improved the plaque burden without impacting the circulating lipids, such as the β-elemene and cacao polyphenols [228,235].

The polyphenols in these treatments were hypothesized to reduce atherosclerosis by several mechanisms. Star anise, β-elemene, cacao polyphenols, CAPE, and Rb1 decreased inflammation, measured by reduced IL-1β, IL-6 and IL-2, TNF-α, VCAM-1, and ICAM-1. CAPE normalized these factors through the direct inhibition of NF-κB. Several studies including cacao polyphenols, β-elemene, and bee pollen also pointed to the reduction in lipid peroxidation as a primary mechanism. The two curcumin studies showed different mechanisms of action. Zhao et al. reported decreased levels of scavenger receptor class A (SR-A), a protein responsible for LDL internalization, and increased ABCA-1, enhancing the cholesterol efflux. This study supplemented curcumin via gavage to animals in a chow diet. On the other hand, Zou et al. determined that curcumin reduced the cholesterol in circulation and in the aorta through the downregulation of the Niemann pick C1-like-1 (NPC1L1), a transporter responsible for intestinal sterol intake. This study supplemented curcumin in a high fat diet. Bee pollen, in addition to reducing oxLDL, reduced the asymmetric dimethylarginine (ADMA) levels, which promoted NO bioavailability by halting its inhibitory effects on NO synthase [238], and reduced endothelial oxidative stress by inhibiting angiotensin-converting enzyme, thus lowering Ang II. AT reduced SREBP-1, monocyte chemoattractant protein 1, a regulator of monocyte migration and infiltration [239], and C-C motif chemokine ligand 5 (CCL55), which recruits leukocytes to the sites of inflammation [240], while also reducing the serum levels of ROS. Finally, the geniposide treatment lessened plaque accumulation by reducing TNF-α as well as inhibiting the p38 MAPK activity. This effect was attributed to geniposide-reducing miR-101, a microRNA responsible for the regulation of several pathways involved in proliferation, promoting the phosphorylation of MAPK and inhibiting the phosphatase mitogen-activated protein kinase-1 (MKP-1).

Overall, the atheroprotective mechanisms associated with the consumption of many different spices center around improvement in inflammation and cholesterol metabolism/efflux and reduction in oxidative stress through the inhibition of lipid peroxidation and through the deactivation of proinflammatory pathways, including the MAPK cascade.

#### 4.4.3. Tea Polyphenols

Teas are soothing beverages made from steeping boiling water with cured leaves of a variety of plants, including *Camellia sinensis*, which is the plant that provides leaves for one of the most consumed teas, green tea. The Yunnan tea tree is the source of leaves for a popular fermented tea, pu-erh tea, which has been consumed regularly throughout China for its benefits associated with the nervous system [241]. Another popular tea is made from the pseudo-fruit of rose bushes, particularly the Rosa rugosa and Rosa canina varieties, which has been consumed in the past to treat scurvy due to its high vitamin C content [242,243]. These bushes originated in East Asia, but they were brought west to Europe and America. It has grown in popularity due to its many beneficial effects, including its antioxidant properties from vitamin C and its polyphenol content, which includes tannins, anthocyanins, flavonoids, and phenolic acids [244,245]. The polyphenols found in teas are primarily catechins, the most well-known being EGCG, the primary flavonoid in green tea, which has anti-inflammatory and antioxidant properties. The catechins contained in green tea and their specific benefits have been reviewed by Babu et al. [246]. Our review search discovered nine papers utilizing polyphenols found in teas. The specific use of EGCG was implemented in four of the studies [247,248,249,250]. Other catechins that are present in green tea, including (−)-epicatechin(EC), (−)-epigallocatechin (EGC), (−)-epicatechin gallate (ECG), (−)-gallocatechingallate (GCG), and caffeine were used in tandem with EGCG in three studies [251,252,253]. Rosehip [254] and pu-erh tea [255] were other sources of supplementation in two studies. Liao et al. [252] were the only group to analyze both male and female mice, while Cavalera et al. [254] were the only group to use only female mice. Xiao et al. [255] did not disclose the sex of the mice. The remaining six studies used male mice, showing the disparity between sexes. All nine studies were conducted in ApoE^−/−^ mice, so no genotype comparisons can be made. Supplementary Table S4 provides experimental details. All the studies showed reductions in plaque.

Rosehip was the only treatment supplemented into the diet. The tea polyphenols [252], tea catechins [251], EGCG [248], green tea polyphenols [253], and pu-erh tea [255] were administered via drinking water. Wang et al. (EGCG) [249] delivered the treatment through oral gavage. The EGCG studies by Chyu et al. [247] and Wang et al. [250] used IP. EGCG had varying effects on lipid profile, and some studies showed marked improvements in HDL, with reductions in TC and LDL [249,253], while others had no effect on these markers [247,250,251]. Miura et al. showed that, while EGCG had no effect on circulating lipids, TC and TG were reduced in the aorta [251]. One study in EGCG [248] did not assess the lipid profile. Rosehip reduced TC, LDL, and VLDL, but had no effect on HDL or TG [254].

The mechanisms of action for EGCG’s atheroprotective activity were improvement in antioxidant and anti-inflammation status [247,249,250,251]. The inflammatory markers reduced by EGCG were TNF-α and IL-6, while the anti-inflammatory cytokine increased was IL-10, as shown by Wang et al. [249]. Other mechanisms involved the reduction in MMP2 and MMP9 (critically involved in plaque progression by remodeling the extracellular matrix [256]) and extracellular matrix metalloproteinase inducer (EMMPRIN), which promoted a more stable plaque with a thicker fibrous cap [250] and improved the autophagosome flux through the increase in LC3-II and Beclin-1 and concurrent reduction in sequestosome SQSTM1/p62 [253]. Additional mechanisms included increased Jagged-1, a critical ligand involved in vascular development mediated by Notch signaling, which protected against endothelial dysfunction by reducing oxLDL [248], and decreases in c-JUN expression, which is a gene associated with cellular proliferation and apoptosis [247]. Rosehip, on the other hand, reduced atherosclerosis, presumably by increasing the expression of the reverse cholesterol transport genes ABCA-1, ABCG-1, and Scarb-1, thus increasing cholesterol clearance. The rosehip treatment also reduced oxidative stress and increased the NO availability.

Teas and their polyphenols have proven to be powerful in ameliorating atherosclerosis, and with their popularity could be a source of great potential treatment plans for patients. Their benefits are mainly derived from their ability to reduce inflammation, but importantly some have been shown to improve cholesterol efflux and improve plaque stability.

## 5. Conclusions

**Figure 3:**
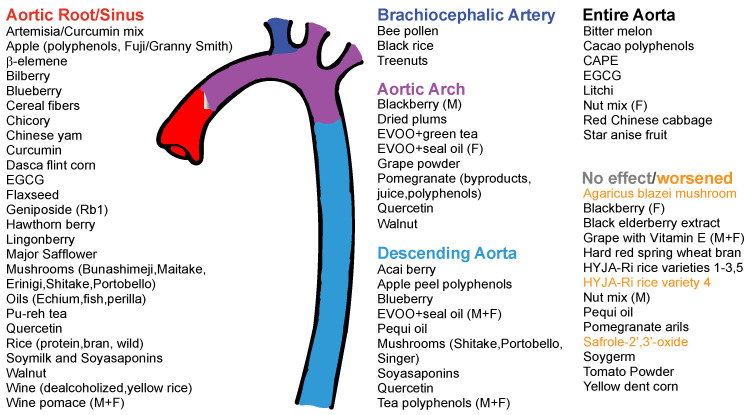
Effect of nutritional interventions in plaque accumulation in the aorta. The effect of different food and extracts in reducing plaque are listed for the aortic arch/sinus, the brachiocephalic artery, the descending aorta, and the entire aorta. Foods that showed no effect and the ones that increased plaque are also listed. Studies using both males (M) and females (F) are also identified.

In conclusion, the studies discussed in this review varied in the use of whole foods and extracts, routes of administration (diet, gavage, drinking water, IP), treatment duration (1 to 24 weeks), diet composition (fat and sugar content of HFDs), and the age of animals. The average age of the animals was 8 weeks, and the average duration of the treatment was ~14 weeks. Despite the differences in experimental design, the majority of the studies reduced plaque. From the studies that reduced plaque, not all changed the lipid profile, and some showed reduced cholesterol in the actual plaque but not in circulation. Approximately 40% of the studies improved the lipid profile with treatment, and approximately 37% did not alter the lipid profile. Only 1% of the studies showed the lipid profile worsening with the treatment. These studies also showed no effect on plaque or even an increase in the plaque burden. The impact on the lipid profile cannot be 100% certain, because several studies did not measure the lipid profiles in the animals. Figure 3 summarizes the effect of different food in different sections of the aortic tree, including the aortic root and sinus, which were analyzed by the majority of the studies; the brachiocephalic artery; the aortic arch; and the descending aorta. Several studies reported a reduction in plaque in the whole aorta, while a few showed no effect and three showed increased plaque. Since the majority of studies only analyzed one section of the aorta, it is unclear whether treatment could be beneficial in another section. Thus, the lack of analysis of the whole aorta is a limitation of these studies and a major gap in this area of research. Another limitation uncovered by this review is the lack of a standardized HFD for atherosclerosis studies, since many studies use HFD with different % of fat and sugar, as listed in the supplementary tables. This makes it difficult to compare the efficacy of each food/extract in different studies.

An additional gap is the low number of studies using females. About 80% of the studies we analyzed were conducted only in male mice. Only six studies looked at both sexes, including one from our group using blackberry [114], the nut mix study [208], the red wine grape pomace [135], the Niagara grape extract with α-tocopherol [133], the EVOO with seal oil [215], and the tea polyphenols [252]. From these studies, the red wine grape pomace and tea polyphenols were effective in reducing plaque in both males and females. It is important to note that the red wine grape pomace treatment only lasted 1–2 weeks and was performed in SR-B1 KO/ApoER61^h/h^ mice. The grape extract with α-tocopherol did not prevent the accumulation of plaque, however it was able to prevent the formation of advanced plaques [133]. Importantly, blackberry [114] was preventative in males but had no effect in females. The opposite was seen with nut mix [208], in which females responded strongly to treatment while males showed no effect. The lipid profile was unaffected by blackberry in both sexes. LDL and VLDL were reduced in both sexes treated with the nut mix; however, only males showed a reduction in TC, and neither sexes saw changes in HDL [208]. EVOO and seal oil significantly reduced plaque in the aortic root and thoracic aorta of female mice, but only reduced plaque in the thoracic aortas of male mice. Surprisingly, both sexes saw improved lipid profiles, suggesting that the mechanism by which EVOO and seal oil impact atherosclerosis is independent of the lipid profile [215].

These findings highlight the importance of including both males and females in nutritional studies. It is also important to note that a reduction in plaque was seen in different sections of the aorta, suggesting that focusing only in the aortic root/sinus by histology may lead to inaccurate conclusions, as mentioned before. Another important consideration when comparing male and female mice is the assessment of hormones, since it is known that estrogens protect females from CVD. Unfortunately, none of the studies using females that we analyzed measured estrogens.

Sex differences in atherosclerosis in several genetic backgrounds are reviewed by Robinet et al. [257]. Some of these differences include larger lesions in female mice compared with male mice. Sex differences in LDLR^−/−^ mice can be found in a review by Mansukhani et al. [258], while the sex differences in ApoE^−/−^ mice are reviewed by Zhang et al. [259]. The differences in the lipid metabolism between the sexes and animal models (ApoE^−/−^ and LDLR^−/−^) should be also considered, as sex differences in the lipid metabolism seem to be linked to gut microbiota, as reported by Baars et al. [260]. Thus, there is a large gap in understanding sex differences in atherosclerosis research using nutritional interventions. Future studies should consider utilizing both sexes in order to determine the effectiveness of the treatment.

This review also highlights the importance of improving the variety of the foods we eat. For example, a diet rich in mushrooms, nuts, berries, apples, olive oil, and tea polyphenols would be effective in reducing atherosclerosis in different sections of the aortic tree and in both sexes (Figure 3).

## Supplementary Materials

The following are available online at https://www.mdpi.com/2072-6643/12/7/2069/s1, Table S1: Fruits. Table S2: Vegetables. Table S3: Nuts and grains. Table S4: Oils, spices, and teas.
