# Supplementary material for: The Impact of Dietary Supplementation of Whole Foods and Polyphenols on Atherosclerosis
Source: Nutrients. 2020 Jul 12;12(7):2069. doi: 10.3390/nu12072069 (PMC7400924; doi:10.3390/nu12072069)
Supplement: Supplementary file 1 [file nutrients-12-02069-s001.pdf]

Table S1. Fruits

Ref	Mouse Model	Treatment	Experimental Design	Treatment Duration	Major Findings	Effect on Lipid profile
113	4-week-old female $ApoE^{-/-}$ mice	Acai berry pulp	AIN-93G with/without 5% freeze-dried Acai pulp	20 weeks	Lesion area in the descending aorta was reduced by 58% compared to control diet. Treatment increased GPx and PON1 activity, and reduced lipid peroxidation, and inflammation.	HDL levels increased with treatment. TC, LDL, and TG did not change.
114	16-week-old male and female $ApoE^{-/-}$ mice	Blackberry	LFD (4.4% fat), HFD (15.9% fat), or HFD with 2% freeze-dried blackberry powder	5 weeks	Blackberry reduced plaque by 57% in the arch in males, but not females. Reduction of Nox1 in males led to decrease in senescence and atherosclerosis.	No effect on male or female in TC, LDL, TG or oxLDL.
115	4-week-old female $ApoE^{-/-}$ mice	Blueberry	AIN-93G diet and 1% freeze-dried blueberry powder	20 weeks	Plaque lesion was reduced 39% in aortic sinus and 57% in the descending aorta. Increased SOD1, SOD2 GSR and Trx1.	TC and LDL cholesterol increased with treatment. No change in HDL or TG.
116	6-week-old male $ApoE^{-/-}$ mice	Lingonberry	LFD (10% kcal), HFD (41% kcal), or HFD with 44% LB	8 weeks	Lesion was reduced in aortic root by treatment, compared to the LFD controls.	TG was reduced, while TC trended towards reduction with treatment.
117	4-week-old male $ApoE^{-/-}$ mice	Hawthorn berry	Standard diet (50 g fat/kg and 1% freeze-dried hawthorn	16 weeks	Hawthorn fruit reduced plaque in the aortic root by 18%. Increased expression of lipid metabolism and antioxidant enzymes (T-AOC, SOD, and GSH-PX).	Reduced TC and TG levels. No effect on HDL.
118	8-week-old male $ApoE^{-/-}$ mice	Bilberry	Standard diet A03 and 0.02% (w/w) anthocyanin-rich bilberry extract (ABE) or fermented bilberry extract (FBE)	16 weeks	FBE reduced plaque in aortic sinus by 36% and ABE reduced plaque by 15%. No mechanism was mentioned however, the extracts are atheroprotective independent of lipid profile and antioxidant statuses.	No effect on TC or TG.

119	6-week-old male ApoE$^{-/-}$ mice	Black elderberry extract (BEE)	AIN-93M diet with/without 0.25% or 1% BEE	24 weeks	BEE did not reduce plaque however, it increased collagen within the plaque making it more stable.	Increased TC, LDL/VLDL, and trended to increase HDL-C with no changes in TG. 0.25% BEE showed no effect on any marker.
133	5-week-old male and female ApoE$^{-/-}$ mice	Grape extract with α-tocopherol	Atherogenic diet (breakdown not provided) with Niagara grape extract in DW, 400 mg/kg tocopherol in diet, and a combination	11 weeks	The diets did not reduce plaque, however the combination diet (α-tocopherol + grape extract) did not show any advanced plaque lesions.	All three diets reduced TC and TG, but the α-tocopherol and grape extract diet resulted in the greatest reduction.
134	6-week-old ApoE$^{-/-}$ mice (gender unspecified)	Grape powder polyphenols	Grape powder dissolved in DW (30 mg/d)	10 weeks	Grape powder reduced lesion size by 41% in the aortic arch with one of the animals not developing lesions at all.	No effect on TC and TG. ox LDL was reduced by 25% and uptake of oxLDL reduced by 33%.
135	8-week-old male and female SR-B1 KO/ApoER61$^{h/h}$ mice	Red wine grape pomace	The pomace was supplemented into a HF (15.9%), high cholesterol (1.25%) and cholic acid-containing (0.5%) diet.	1 or 2 weeks	Reduced plaque accumulation in the aortic root. Reduction of TNF-α and increasing antioxidant capacity of HDL.	No effect on TC, and improved antioxidant activity as measured by HDL-containing plasma and DHR oxidation.
136	4-week-old male ApoE$^{-/-}$ mice	Dealcoholized red and white wines	25 ml of dealcoholized red or white wine/kg BW/d mixed into the diet (0.15% cholesterol)	12 or 20 weeks	Red (62%) and white (30%) wine reduced plaque in the thoracic aorta, but only red wine reduced plaque in the aortic root (16%). Reduction of VCAM-1, ICAM-1, and pathways involving NF-κB, PI3K, IFN-1, and IL-1β.	No effect on lipid profile.

137	7-week-old male LDLR$^{-/-}$ mice	Yellow rice wine (polyphenol content similar to red wine)	HFD (10% fat) with 10, 30, or 50 mg/kg/d of yellow rice wine polyphenols in DW	14 weeks	The strongest reduction in plaque in the aortic root resulted from 30 mg/kg/day at 40.09%, but all treatments caused significant reductions. Reductions in MMP2 and MMP9 and increases in TIMP1 and TIMP2.	All three treatment doses reduced TC and LDL. TG tended to reduce with treatment, but it was not significant. No effect on HDL.
138	16-week-old male ApoE$^{-/-}$ mice	Pomegranate juice (PJ)	Standard diet with/without PJ (~31 μL/d) in DW	8 weeks	PJ did reduce lesion size in the aortic arch compared to a placebo.	No effect on TC, but PJ increased serum paraoxonase activity by 26%.
139	6-week-old male ApoE$^{-/-}$ mice	Pomegranate byproduct (PB)	Standard chow diet with/without PB diluted in DW with 17 or 51 gallic acid	12 weeks	PB reduced lesions in the aortic arch and increased PON2 activity which lead to decreased ox-LDL uptake by macrophages, reducing oxidative stress.	Serum TC/TG were not assessed. Macrophage cholesterol content was reduced. GSH and PON2 were increased with PB
140	6-week-old ApoE$^{-/-}$ mice (gender unspecified)	Pomegranate peels, arils, flowers, and whole juice	All pomegranate extracts were given in 200 μg of gaellic acid equivalents/mouse/d	12 weeks	All treatments reduced lesion size in the aortic arch, with pomegranate flower extract causing the highest reduction (70%). Reduced oxidative stress in macrophages, serum lipids and lipid peroxidation.	Flower extract reduced serum TC, TG, and glucose levels. Aril extract reduced TG. No other extracts impacted lipid profile.
148	9-week-old ApoE$^{-/-}$ male mice	Cider apple polyphenol extracts, fiber, or both	AIN-93G with 16,7% apple fiber extract, 0.75% polyphenol extract, or both.	16 weeks	All three groups showed reductions in plaque in the aortic sinus, the fiber and combination treatments were similar (38.3 and 38.6% reduction) and polyphenols only led to 16.5% reduction.	No effect on plasma TC or TG, but the combination treatment reduced hepatic cholesterol levels.
149	4-week-old ApoE$^{-/-}$ male mice	Granny Smith apple peel	Normal diet (1.8% fat), or HFD (17.6% fat) w/without healthy apple peel (20% w/w)	10 weeks	Apple peel reduced plaque in the thoracic aorta and cholesterol content within the plaque potentially due to antioxidant effects. Apple peel also lowered fibrosis.	TC was unchanged with apple peel but triglyceridemia was brought back to control levels

150	12-week-old male ApoE$^{-/-}$ mice	Apple polyphenols	Western-type diet (21% fat) with apple polyphenols (100 mg/kg/d) by oral gavage	12 weeks	Apple polyphenols reduced plaque in the aortic sinus and inflammatory pathways involving NF-κB, MAPK, leading to reduced VCAM-1 and CCL2 levels.	HDL was increased, while TC, TG, and LDL were significantly reduced.
151	6-week-old male ApoE$^{-/-}$ mice	Litchi pericarp procyanidins (LPPC)	AIN-93G (20% lard) with/without litchi pericarp procyanidins in DW 100 mg/kg/day (water prepared daily)	24 weeks	LPPC reduced plaque in the entire aorta by 23%, while reducing HMG-CoA and increasing ABCA1	Lipid profiles were not assessed. LPPC lowered NO levels, iNOS activity, and TBARS content in plasma. SOD activity was increased.
152	5-week-old male ApoE$^{-/-}$ mice	Dried Plums (prunes) (DP)	AIN-93G with low (4.5%) or high (9.5%) DP	20 weeks	The low DP diet produced significant decreases in lesion area in the arterial trees/aortic arch, but the high DP diet only showed trends toward reductions.	DP did not change TC or TG

Table 2. Vegetables.

Ref	Mouse Model	Treatment	Experimental Design	Treatment Duration	Major Findings	Effect on Lipid profile
157	6-week-old male ApoE$^{-/-}$ mice	Anthocyanins from purple sweet potato	AIN-93G (15% lard) with/without 1% APSP	4 weeks	Purple sweet potato reduced plaque throughout the entire aorta and improved oxidative stress/inflammatory markers, independent of cholesterol level. Reduced TNF-α-induced inflammation and VCAM-1 and ICAM-1 expression.	No significant effect on lipid profiles
158	4-week-old male ApoE$^{-/-}$ mice	Chinese yam extracts (β-sitosterol and ethyl linoleate)	Western diet (AIN-76A, 40% fat) and 200 mg/kg BW/d of extracts by gavage 3 d/w	11 or 21 weeks	Plaque and lipids in the aortic root were reduced with yam extracts. Increased macrophage content of plaques and reduced IL-6, VCAM-1	Yam extracts significantly reduced TC, oxLDL, and CRP in plasma. HDL trended to increase with treatment.

					expression, lipid profile, and CRP levels.	
159	8-week-old male ApoE$^{-/-}$ mice	Bitter melon	HFD (21.8% fat) with/without bitter melon (1.2% w/w)	16 weeks	Plaque size in the entire aorta was reduced, and plaque composition showed trending reductions of VSMCs, macrophages, and collagen. Reduction in VCAM-1 is the proposed mechanism.	Treatment reduced TG levels however, no other changes were observed in the lipid profile.
163	16-week-old male ApoE$^{-/-}$ mice	Chicory	AIN-93 diet with/without 5 g/kg freeze-dried chicory	10 weeks	Chicory reduced plaque in aortic sinus by 39% and increased ABCA1 and ABCG1 expression.	Treatment reduced cholesterol in the aortic root and did not affect blood lipid profile
164	14-week-old male ApoE$^{-/-}$ mice	Anthocyanins from red Chinese cabbage (ArCC)	Western diet (21.2% fat) with/without low (16 mg/kg) or high (300 mg/kg) ArCC by gavage	12 weeks	ArCC treatment strongly reduced plaque in the whole aorta by limiting expression of adhesion molecule VCAM-1 and though improved antioxidant capacity and lipid metabolism.	High dose of ArCC lowered LDL, VLDL, and TC. The low dose ArCC only lowered LDL and VLDL.
165	6-week-old male ApoE$^{-/-}$ mice	Quercetin	AIN-93M with/without quercetin or theaflavin (1.3 mg/d)	20 weeks	Quercetin and theaflavin reduced plaque in the aortic sinus and thoracic aorta, but quercetin was more effective. Improved NO availability, heme-oxygenase-1 activity, and reduced inflammatory LTB_4.	No effect on TC or lipoprotein distribution.
166	4-week-old male ApoE$^{-/-}$ mice	Quercetin	HFD with/without 0.05% (w/w) Quercetin (1.5 mg/g)	14 weeks	Reduced lesion thickness in the aortic arch and thoracic aorta, and increased eNOS activity and heme-oxygenase-1.	Quercetin reduced TC and TG.
172	5-week-old male ApoE$^{-/-}$ mice	Soyasaponin A1 (A1) or A2 (phytochemicals from soybeans)	HFD (45% fat) with or without 10 or 20 µmol kg-1 Soyasaponin A1 or A2	24 weeks	Both Soyasaponin A1 and A2 significantly Reduced plaque in the aortic root and descending aorta, and decreased hypercholesterolemia and inflammation due to increased	A1 reduced TG, LDL, TC and increased HDL. A2 resulted in similar results but did not affect HDL cholesterol.

					cholesterol efflux and reduced TNF-α and MCP-1.	
173	8-week-old male ApoE$^{-/-}$ mice	Soygerm	Western diet (23.2% fat) with/without 10% tomato powder, 2% soy germ, or a combination	4 weeks	Neither had effect on atherosclerotic plaque.	Both treatments increased TC levels and had no effect on TG.
174	12-week-old male LDLR$^{-/-}$ mice	Soymilk	Hyperlipidic diet (20% fat, 1.25% cholesterol, 0.5% cholic acid) with/without soy milk (0.6 mL) by gavage	8.5 weeks (60 days)	Soymilk reduced plaque in the aortic root, and reduced hyperlipidemia, CRP and CD40L and collagen deposition.	Reduced TCL, LDL, VLDL, and TG. Both VLDL and TG were reduced back to basal levels in control animals. HDL was increased.
181	12-week-old male ApoE$^{-/-}$ mice	*Grifola gargal* Singer mushroom	Ang II via osmotic minipump and 10 mg/kg BW of *Grifola gargal* extract by IP twice weekly	4 weeks	Reduction of thoracic aorta plaque and circulating granulocytes and normalization of Tregs which inhibit excessive response of T Cell subtypes, Th1 and Th17.	Not assessed.
182	4-week-old male LDLR$^{-/-}$ mice	Portobello and shiitake mushrooms	HFD (18.9% fat) with 10% (w/w) portobello or shiitake mushrooms	16 weeks	Mushrooms reduced lesion by 86% (shiitake) and 70% (Portobello) in the descending aorta and aortic sinus. Reduced TNF-α, lower circulating lipids, and reduced VCAM-1 expression.	Both reduced TC, TG, and LDL. Portobello had no effect on HDL and Shiitake decreased HDL.
183	6-week-old male ApoE$^{-/-}$ mice	*Pleurotus eryngii* (Eringi), *Grifola frondosa* (Maitake), and *Hypsizygus marmoreus* (Bunashimeji) mushrooms	Normal diet (<66 mg/100 g cholesterol) with 3% of Erinigi, Maitake, or Bunashimeji mushrooms	10 weeks	All three mushrooms reduced plaque in the aortic root. Bunashimeji had the most impact on plaque. Improved lipid profile which was independent of cholesterol absorption. It is predicted that it may be through enhanced fecal excretion, but further tests are needed.	Erinigi and Maitake had no effect on TC except for week 6 of diet, but Bunashimeji lowered TC. TG was not affected by any treatment.

184	ApoE$^{-/-}$ mice (age, gender unspecified)	*Agaricus blazei* Mushroom	AIN-93M diet with/without 5% powdered *Agaricus blazei*	6 or 12 weeks	Increase lesion size in the aortic valve and reduced collagen leading to less stable plaques. Increased MMP9 and IFN-γ. Increased stimulation of NKT cells and circulating neutrophils and macrophages.	There was no effect on lipid profile.

Table S3. Nuts and Grains

Ref	Mouse Model	Treatment	Experimental Design	Treatment Duration	Major Findings	Effect on Lipid profile
192	4-week-old male LDLR$^{-/-}$ mice	Dasca-flint Corn fractions (aleurone, germ, and endosperm)	Mouse Diet 9F (8.5% fat, 0.06% cholesterol) with 5% (w/w) aleurone, endosperm, or germ	10 weeks	Aleurone and germ decreased plaque in the aortic root. Endosperm tended to reduce plaque, but it was not significant. Reduced in serum TC and LDL in increased fecal lipid excretion.	Aleurone and germ corn reduced TC but had no effects on HDL, LDL or VLDL. Endosperm had no effect on any lipid profile. No effect on TG.
193	30-week-old male ApoE$^{-/-}$ mice	Anthocyanins from black rice	AIN-93G with either 50 mg/kg/d simvastatin or 300 mg/kd/d of black rice anthocyanin-rich extract	20 weeks	Anthocyanin increased thickness of the fibrotic cap and decreased the size of necrotic core in brachiocephalic arteries. Decreased MMP1, TF, and, iNOS.	Anthocyanins lowered TC, LDL, VLDL, TG, and HDL.
194	7-week-old male ApoE$^{-/-}$ mice	Red Yeast Rice (RYR)	Three groups, normal diet, HFD (15%) with saline, HFD with RYR (0.34g kg/day)	12 weeks	RYR reduced plaque area in the ascending aorta by 15% potentially through improved lipid metabolism and gut microbiota status, and reduced inflammation.	Treatment reduced TC and LDL cholesterol. No changes in TG.
195	7-week-old male ApoE$^{-/-}$ mice	Rice Bran Enzymatic Extract	HFD (42%) with/without 5% (w/w) rice bran enzymatic extract	23 weeks	Reduced plaque area in the aortic sinus as well as macrophage infiltration. Enhanced apoptosis of spleen mononuclear cells and improved lipid profile.	Reduced TC, TG, and increased HDL.

196	4-week-old female $ApoE^{-/-}$ mice	Rice Protein Isolate (RPI)	AIN-93G diet with/without casein or RPI as the source of protein (~ 18%)	16 weeks	Lesion was reduced by 55% in the aortic sinus and 40% in the aortic tree. Improved antioxidant capacity caused by increases in CAT, SOD-1, GSR, GPX1, and GPX3.	No effect on TC and HDL and reduced ox-LDL and anti-ox-LDL IgG levels.
197	4-week-old male $LDLR^{-/-}$ mice	Wild Rice	AIN-93G diet (0.06 cholesterol) with either 60% (w/w) carbs from wild rice, 2% (w/w) phytosterol mixture, or both	20 weeks	All three treatments reduced plaque in the aortic root. The combination treatment showed almost no plaque. No specific mechanism proposed but theorized that it involves the dietary fiber and phenolic compounds found in the treatments.	2% phytosterols and combination treatment increased total lipids in fecal samples. Other lipid profile markers were not assessed.
198	6-week-old $ApoE^{-/-}LDLR^{-/-}$ mice (gender unspecified)	HYJA-Ri rice variety variants 1-5	Western diet (20% fat) with/without rice (39.7%)	12 weeks	1, 2, and 5 did not change plaque, 3 slightly increased, 4 significantly increased plaque.	Not assessed.
199	37-week-old male $ApoE^{-/-}$ mice	Cereal Fibers (oat and wheat bran)	High Fat/Cholesterol (46% fat) diet with either 0.8% oat or wheat bran fibers	18 weeks	Plaque was reduced in the aortic root by oat fiber (43.3%) and wheat bran (27.1%). Reduced chronic inflammatory response from pathways with NLRP3 inflammasome and TLR4/MyD88/NF-κB, and inhibition of foam cell formation.	Oat fiber reduced TC and LDL, wheat bran tended to reduce both, but neither were significant. Neither had effects on TG or HDL.
200	4-week-old male $ApoE^{-/-}$ mice	Yellow dent corn and hard red spring wheat bran	AIN-93G with/without 1.7% corn bran or 3.3% wheat bran	18 weeks	No effect on plaque from either treatment	No effect on any lipid profile markers
201	6-7-week-old male $ApoE^{-/-}$ mice	Major Safflower	AIN-93G and 1.0% safflower extract, 0.1% N-(p-Coumaroyl)-	15 weeks	Reductions in plaque at the aortic sinus are attributed to the reduced lipid peroxidation and TC.	TC was significantly reduced.

			serotonin, or N-Ferulosylserotonin			
206	8-week-old male ApoE$^{-/-}$ mice	Walnut	HFD (34% fat) with either walnut oil or homogenized walnut (1.2 g/5 g diet)	8 weeks	Whole walnut, but not walnut oil, reduced plaque (55%) in the aortic arch.	Whole walnut reduced TG and TC, walnut oil had no effect on lipid profile
207	10-12-week-old male ApoE$^{-/-}$ mice	Tree nuts (Macadamia and Pecan)	Ang II in minipump, standard diet (4.8% fat) and tree nuts (2.5% macadamia. 2.5% pecan: 5.75 g/kg/day)	8 weeks	Tree nuts reduced plaque in the brachiocephalic artery, but not in the aortic arch.	Not assessed.
208	8-12-week-old male and female ApoE$^{-/-}$ mice	Nut mix (50% walnut, 25% almond, 25% hazelnut)	Standard chow diet (5-8% fat) diet with 3% (w/w) nut mix or 2% (w/w) palm oil	12 weeks	The nut mix reduced plaque in the entire aorta in females, but not in males.	Reduced VLDL and LDL cholesterol in both genders and reduced TC in males, but not females. No effect in HDL in either genders or groups.
209	5-7-week-old female LDLR$^{-/-}$ mice	Flaxseed	Chow diet with/without 10% ground flaxseed or atherogenic diet (2% cholesterol) with either 1%, 5%, or 10% ground flaxseed	24 weeks	10% ground flaxseed reduced plaque in the aortic arch. 5% tended to reduce plaque but it was not significant. 1% had no effect on plaque. Mechanism of action likely surrounds cholesterol-lowering effect or reduction in IL-6, mac-3, VCAM-1, or PCNA.	Flaxseed reduced TC and plasma saturated fatty acids. No other profile markers were assessed.

Table S4. Oils, Spices, and Teas

Ref	Mouse Model	Treatment	Experimental Design	Treatment Duration	Major Findings	Effect on Lipid profile
214	6-week-old male ApoE$^{-/-}$ mice	Extra Virgin Olive Oil with Green Tea Polyphenols (EVOO-GTPP)	Diet not mentioned. Mice received 7 μL/day of EVOO or EVOO-GTPP by gavage	8 weeks	Treatments reduced plaque by 20%, (EVOO-GTPP) and 11% (EVOO) in the aortic arch. Decreased oxidative stress and improved macrophage cholesterol metabolism and HDL-mediated cholesterol efflux	EVOO-GTPP reduced ox-LDL and lipid peroxidation other markers not assessed.
215	6-week-old male and female ApoE$^{-/-}$	Extra Virgin Olive oil (EVO) and Seal oil	HFD (19.5% fat, 1.25% cholesterol) with/without 1% (w/w) EVO and seal oil or 1% corn oil	12 weeks	Treatment reduced plaque in the aortic arch and descending aorta in female mice and only in the descending aorta in males	Plasma TC levels were reduced in both male and female mice.
216	6-8-week-old female LDLR$^{-/-}$	Pequi oil	AIN-93G with/without 7% pequi oil	6 weeks	Increased lesions in the aortic root and reduced lesions in the thoracic aorta. Reduction due to antioxidant properties of the oil. Fatty acids in the oil may cause increased plaque in the aortic root.	Pequi oil increased TC, LDL/VLDL, and TG but reduced oxLDL. HDL was unaffected by treatment.
217	6-week-old male ApoE$^{-/-}$ mice	Perilla oil vs. Sunflower oil	Paigen's atherogenic diet with 10% perilla oil, sunflower oil, or lard	10 weeks	Perilla oil, but not sunflower oil, reduced lesion in the aortic sinus. Possible mechanisms include increasing eNOS, reducing iNOS, and VCAM-1/ICAM-1, and normalizing lipid profile.	Perilla oil reduced TG, TC, and LDL and increased HDL. Sunflower oil reduced TG, TC, and LDL, but it had no effect on HDL.
218	6-8-week-old female LDLR$^{-/-}$ and ApoE$^{-/-}$ mice	Palm (PO), Echium (EO), and Fish oil (FO)	Atherogenic diets (20% cal as fat) with 10% cal from PO and additional 10% from either PO, EO, or FO.	LDLR$^{-/-}$: 16 weeks ApoE$^{-/-}$: 12 weeks	Treatment reduced plaque the aortic root of LDLR$^{-/-}$ mice, no effect in ApoE$^{-/-}$ mice	EO and FO significantly reduced VLDL and LDL, and increased HDL in LDLR$^{-/-}$ mice. No effects of EO or PO in ApoE$^{-/-}$ mice. FO increased TC.

226	16-week-old male ApoE$^{-/-}$ mice	Curcumin	Chow diet (4.5% fat) with curcumin (20mg/kg body weight) or saline via gavage	4 weeks	Reduced plaque in aortic roots potentially due to inhibition of macrophage cholesterol uptake by SR-A and efflux by ABCA1.	TC, TG, and LDL/VLDL were reduced by curcumin
227	8-week-old male ApoE$^{-/-}$ mice	Curcumin	HFD (41% fat) with/without 0.01% w/w curcumin	16 weeks	Reduction in aortic sinus plaque associated with reduced cholesterol through the downregulation of the Niemann pick C1-like-1 (NPC1L1) transporter,	TC and LDL were reduced and HDL was increased. TG was not affected.
228	8-week-old ApoE$^{-/-}$ mice (gender unspecified)	Cacao polyphenols (catechin, epicatechin, procyanidin B2/C1, and cinnamtannin A2)	AIN-93G diet with/without 0.25 or 0.4% cacao polyphenols in the diet.	24 weeks	0.4% cacao had the strongest effect on plaque in the whole aorta (down 4% from control). Reduction can be attributed to the decrease in VCAM-1, ICAM-1, 4-hydroxynonenal, hexanoyl-lysine, and dityrosine.	No effect on TC, HDL, or TG levels.
229	6-week-old male ApoE$^{-/-}$ mice	*Artemisia iwayomogi* and *Curcuma longa* (turmeric) (AT)	Western diet (21% fat) with/without 50, 100, or 200 mg/kg of AT or 50 mg/kg of curcumin in diet	10 weeks	Plaque was reduced in the aortic root by AT treatment most likely due to the restoration of normal SREBP-1c, FAS, SCD-1, PPAR-α, CPT-1, IL-6, IL-1B, and TNF-α levels.	AT treatment lowered TC, LDL, and TG, and improved TC/HDL ratio as well as HDL levels.
230	4-week-old female ApoE$^{-/-}$ mice	Bee pollen	HFD (21% lard) with/without extract of bee pollen (0.1 or 1 g/kg)	16 weeks	Plaque was partially (0.1 g/kg) or completely (1 g/kg) reduced by pollen extract in the brachiocephalic artery.	0.1 and 1 g treatments significantly reduced TC and oxLDL.
231	6-week-old male ApoE$^{-/-}$ mice	*Illicium verum* (Star Anise) fruit	HFD (45% fat) with either 100 mg/kg or 200 mg/kg star anise	12 weeks	Plaque was reduced in the entire aorta due to reductions in TNF-α, IL-1β, NF-κB, COX and adhesion molecules in the aorta (VCAM-1 and ICAM-1).	TC and LDL were reduced, HDL and TG were unchanged.

233	10-week-old male $ApoE^{-/-}$ mice	Safrole-2′,3′-oxide (SFO)	Atherogenic diet (0.25% cholesterol) for 10 weeks then treatment with SFO (50 or 100 mg/kg/d) by IP 4 d/w	4 weeks	50 and 100 mg increased plaque. Additionally, plaques in SFO treated mice were less stable due to increases in MMP levels.	TC, TG, and LDL were increased and HDL was unchanged.
234	10-week-old male $ApoE^{-/-}$ mice	Caffeic acid phenethyl ester (CAPE) (from bee propolis)	CAPE was supplemented into the diet (30 mg/kg BW) (diet information not provided)	12 weeks	CAPE significantly reduced lesions in the thoracic aorta through the reduction in inflammation markers due to the inhibition of NF-κB.	No effect on lipid profile.
235	4-week-old male $ApoE^{-/-}$ mice	β-elemene (from *Curcuma Wenyujin*)	HFD (20% fat, 2% cholesterol) for 4 weeks. Then HFD or HFD with β-elemene 135 mg/kg) administered by gavage for 12 weeks	16 weeks	Plaque was reduced in the aortic sinus in part due to reduction in lipid peroxidation and inflammation markers including mac-3 and ROS, and improvements in GSH, SOD, GPx, and catalase.	No effect on lipid profile.
236	6-week-old male $ApoE^{-/-}$ mice	Ginsenoside Rb1 (from Chinese ginseng)	Western diet (21% fat) with Rb1 (10 mg/kg/day) or saline IP from weeks 4-12.	12 weeks	Rb1 reduced plaque in the aortic sinus and IL-1β, IL-6, and TNF-α, and by induced autophagy with decreased p62 and increased LC3 and Beclin-1.	RB1 significantly reduced TC, TG, and LDL cholesterol.
237	8-week-old $ApoE^{-/-}$ mice (gender unspecified)	Geniposide (from *Gardenia jasminoides Ellis* fruit)	HFD (21% pork lard) with/without geniposide (50 mg/kg/d) by gavage	12 weeks	Significantly reduced plaque in the aortic sinus by reducing miR-101 leading to inhibition of p38 and reductions in TNF-α.	Significant reductions in blood glucose, TC, and LDL. HDL and TG were not affected.
247	7-week-old male $ApoE^{-/-}$ mice	Green Tea-derived Catechin (EGCG)	HFD (21% fat) with/without EGCG (10 mg/kg) by IP 5 d/w.	3 or 6 weeks	EGCG reduced plaque at earlier stages of atherosclerosis compared to mature stages. Reduced superoxide	No effect on plasma cholesterol levels.

					and TNF-α and c-Jun, and increased AP-1 activation.	
248	6-week-old male ApoE$^{-/-}$ mice	Epigallocatechin-3-gallate (EGCG)	HFD with/without 0.8 g/L EGCG in DW	15 weeks	EGCG reduced plaque progression through increased Notch/Jagged-1 signaling and HES5 expression.	Not assessed.
249	7-week-old male ApoE$^{-/-}$ mice	EGCG from green tea	HFD with/without EGCG (40 mg/kg/day) dissolved in distilled water via gavage	18 weeks	EGCG reduced plaque potentially due to reductions in inflammatory markers (TNF-α and IL-6) and increase of IL-10.	Dramatic increases in HDL levels were accompanied by decreases in TC and LDL. No effect on TG.
250	8-week-old male ApoE$^{-/-}$ mice	Epigallocatechin-3-gallate (EGCG)	HFD (21% fat) with EGCG (10 mg/kg BW 5 d/w) or saline (0.9%) by IP	16 weeks	EGCG reduced plaque and its macrophage content. Collagen and VSMC deposition in the plaques was increased. Increased thickness of the fibrotic cap.	EGCG did not alter TC, TG, LDL, or HDL-cholesterol.
251	8-week-old male ApoE$^{-/-}$ mice	Green Tea Polyphenols (GTP)	HFD (12% lard) with/without GTP (3.2 or 6.4 g/L) in DW	15 weeks	GTP reduced lipid droplets and plaque in the aortic root. Increased LC3-II and Beclin-1 and reduced p62/SQSTM1.	Reduced TC, LDL, oxLDL, and TG in the liver and plasma and increased HDL.
252	10-week-old male ApoE$^{-/-}$ mice	Tea Catechins (EGCG, EGC, EC, GCG, ECG, and caffeine)	Atherogenic diet (1.25% cholesterol) with/without 0.8 g/L tea extract in DW with 30 g/L sucrose	14 weeks	Plaque in the arch to the femoral bifurcation/aortic weights were reduced, hypothetically through improvement in antioxidative activity.	Plasma TC and TG not affected by catechins, but aortic TC and TG were significantly reduced.
253	8-week-old male and female ApoE$^{-/-}$ mice	Tea Polyphenols (TP)	HFD (15% fat) with/without either 1.6 g/L, 0.8 g/L, or 0.4 g/L TP in DW with 30 g/L sucrose	16 weeks	All TP doses reduced plaque in the abdominal aorta, the 0.4 g/L showed the weakest effect. Increased *Bifidobacteria pseudolongum* in the intestines, leading to improved fat metabolism.	No effect on TG levels. 0.4 g/L had no effect, while 0.8 ad 1.6 g/L significantly reduced TC, LDL, and HDL.

254	8-week-old female ApoE-/- mice	Rose hip	HFD (45% energy from fat) with/without rose hip (303.8 g/kg)	24 weeks	Rose hip reduced plaque throughout the aorta and proinflammatory markers (ox-LDL and fibrinogen). Plaque reduction attributed to improved cholesterol efflux.	Reduced TC and LDL/VLDL but HDL was unchanged. Decreased TC/HDL cholesterol ratio.
255	6-week-old ApoE-/- mice (gender unspecified)	Pu-reh tea	Western diet (21% fat) with pu-reh tea in DW (concentration not specified)	8 or 16 weeks	Plaque at aortic root was reduced in 8-week group. Reduced macrophages in the plaque caused by increased macrophage apoptosis. Reduced NF-kB and TNF-α.	No effect on TC or TG or oxLDL.

Abbreviations: TC (total cholesterol), TG (triglycerides), LFD (low fat diet), HFD (high fat diet), DW (drinking water), d (day), BW (body weight), T-AOC (total antioxidant capacity), SOD (superoxide dismutase), and GSH-PX (glutathione peroxidase).
